# Thiol–Ene Click-Derived Porous-Layer Open-Tubular Column for Online Purification and Multiplex LC–MS/MS Analysis of Neurotransmitters

**DOI:** 10.1021/acs.analchem.6c04601

**Published:** 2026-08-24

**Authors:** Enrico Taglioni, Andrea Cerrato, Aldo Laganà, Carmela Maria Montone, Anna Laura Capriotti

**Affiliations:** † Department of Chemistry, 9311 Sapienza University of Rome, Piazzale Aldo Moro 5, 00185 Rome, Italy; ‡ Interuniversity Consortium INBB − Biostructures and Biosystems National Institute, Via dei Carpegna 19, 00165, Rome, Italy

## Abstract

A thiol–ene-based porous stationary phase (PLOT-type coating) was developed for online sample cleanup and enrichment coupled to LC–MS/MS for the simultaneous determination of structurally diverse neurotransmitters in biological matrices. The resulting porous thiol–ene functionalized capillary column combines the inherently high permeability of the open-tubular architecture with tailored surface functionality, enabling direct injection and efficient handling of highly polar, low-abundance analytes without derivatization. The method allows multiplexed quantification of serotonin, dopamine, glutamic acid, γ-aminobutyric acid, and reduced glutathione in plasma, cell samples, and brain tissue within a simplified analytical workflow. Compared to conventional off-line protocols, the proposed approach minimizes sample preparation while maintaining high sensitivity and reproducibility, with recoveries ranging from 86% to 100% and matrix effects within 90–104%. The platform demonstrates reliable analytical performance across different biological matrices and is compatible with routine LC–MS/MS instrumentation. Overall, this work establishes the thiol–ene-porous capillary as a viable strategy for integrated sample processing in bioanalysis and expands its application to polar metabolite profiling in complex systems.

## Graphical abstract

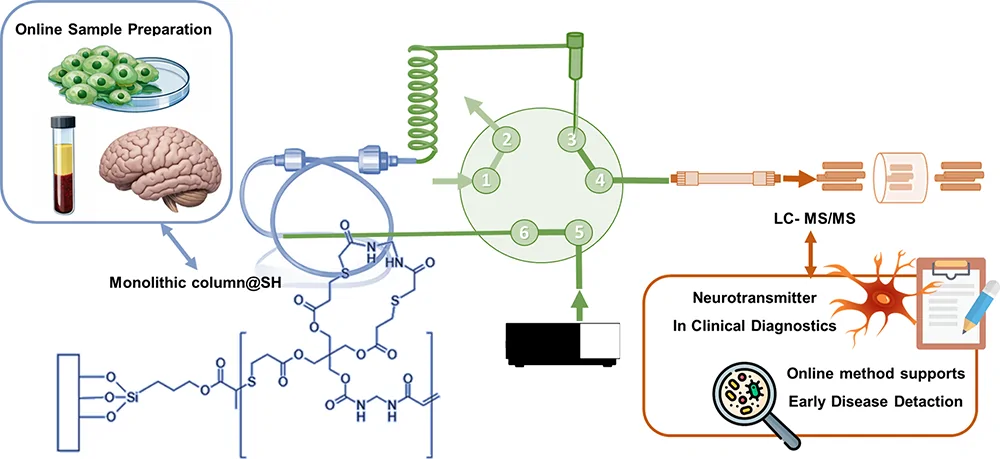

## Introduction

Accurate quantification of neurotransmitters remains a significant analytical challenge due to their low endogenous concentrations, high polarity, and the complexity of biological matrices. These small bioactive molecules are involved in cellular signaling and redox regulation and are highly relevant in physiological, pharmacological, and clinical studies.^1^ Liquid chromatography coupled with tandem mass spectrometry (LC–MS/MS) is widely employed for targeted neurotransmitter analysis because of its sensitivity, selectivity, and multiplexing capabilities.^2,3^ Several LC–MS/MS methods have been reported for the determination of neurotransmitters in complex biological matrices such as mouse brain tissue and cerebrospinal fluid.^2–7^ Despite these advances, robust strategies for the derivatization-free and multiplexed quantification of neurotransmitters in accessible matrices such as plasma and cultured cells remain limited. In plasma, neurotransmitters are typically present at ng mL^–1^ or pg mL^–1^ levels within highly complex matrices containing proteins, lipids, salts, and endogenous metabolites that can interfere with detection. Consequently, conventional workflows often rely on multistep sample preparation procedures, including protein precipitation, solvent extraction, and derivatization prior to LC–MS/MS analysis.^8–12^ These approaches increase analysis time, reduce throughput, and may introduce variability, highlighting the need for more streamlined and robust analytical strategies. To overcome efficiency limitations, porous-layer open-tubular (PLOT) capillary columns, consisting of a porous layer deposited on the inner wall of the capillary, have been extensively investigated in gas chromatography (GC) and later adapted to capillary electrochromatography (CEC) and liquid chromatography (LC) because they combine high permeability, low backpressure, and rapid mass transfer.^13,14^ More recently, these columns have also been implemented in liquid chromatography (HPLC) as trapping columns.^15^ The development of PLOT columns has historically relied on the deposition or in situ synthesis of stable adsorbent layers, including porous polymers, alumina, and silica, to maximize surface area while maintaining low flow resistance.^16^ Among these materials, porous polymer-based layers have attracted particular interest owing to their facile synthesis, tunable morphology, and suitability for microscale separations.^17^ In this context, click chemistry has emerged as a powerful strategy for stationary-phase fabrication because it enables efficient, orthogonal, and highly controllable polymerization and postfunctionalization reactions. Early studies, including the pioneering work of Liu et al., demonstrated the applicability of click-polymerized monoliths for liquid chromatography and established the foundation for the development of thiol–ene-derived stationary phases tailored to specific separation challenges.^18^ Among these approaches, thiol–ene click-derived porous polymer stationary phases have recently gained attention as versatile materials for analytical separations due to their high permeability, tunable porosity, and modifiable surface chemistry.^19^ While previous studies have employed thiol–ene monoliths for off-line enrichment of polycyclic aromatic hydrocarbons via reversed-phase interactions^20^ or for trace-level extraction of estrogens from complex matrices through combined π–π and hydrophobic interactions,^21^ these approaches did not address the challenges associated with online preconcentration. Here, we present an online enrichment strategy coupled directly to LC–MS/MS for the analysis of highly polar, low-abundance neurotransmitters in biological fluids. The synthesized capillary column was designated as TE-PLOT, referring to a thiol–ene porous layer open-tubular stationary phase, in which a thin, porous polymeric network is covalently anchored to the inner wall as a coating, leaving an open central channel. By utilizing this PLOT configuration rather than a fully packed monolithic column, the system achieves specific interactions with neurotransmitters, enabling improved selectivity and sensitivity compared to previous offline protocols. The proposed approach enables derivatization-free, multiplexed determination of key neurotransmitters, including serotonin (5-HT), dopamine (DA), glutamic acid (Glu), reduced l-glutathione (GSH), and γ-aminobutyric acid (GABA), in peripheral blood and other biological matrices. The integration of the IT-SPME step provides simultaneous sample cleanup and enrichment, which facilitates neurotransmitter determination in complex biological matrices and reduces sample handling and matrix-related interferences.

## Materials and Methods

### Materials

HPLC-grade solvents, all reagents used for sample preparation, sodium hydroxide (NaOH), hydrochloric acid (HCl), 3-(trimethoxysilyl)­propyl methacrylate (TMSPMA), *N*,*N*′-methylenebis­(acrylamide), diethylene glycol diethyl ether (DGDE), 2,2′-azobis­(2-methylpropionitrile) (AIBN), poly­(ethylene glycol) with an average molecular weight of 200 (PEG), and pentaerythritol tetrakis­(3-mercaptopropionate) (PETMP) were obtained from Merck Life Science (Darmstadt, Germany). Fused silica-lined PEEK tubing (PEEK-sil, 150 × 0.530 mm I.D. and 1.59 mm O.D.) was purchased from SGE Analytical Science (Palmer, USA). Analytical standards of serotonin (5-HT, CAS No. 50–67–9), dopamine (DA, CAS No. 62–31–7), glutamic acid (Glu, CAS No. 56–86–0), reduced l-glutathione (GSH, CAS No. 70–18–8), and γ-aminobutyric acid (GABA, CAS No. 56–12–2) were supplied by Merck Life Science (Darmstadt, Germany). Individual stock solutions were prepared in ultrapure water at a concentration of 1 g L^–1^ using certified reference materials. Working solutions containing all analytes at a final concentration of 2 μg mL^–1^ were obtained by appropriate dilution with water. These solutions were aliquoted and stored at −20 °C to prevent repeated freeze–thaw cycles. Plasma samples were obtained from 5 mL of whole human blood collected from five healthy volunteers after written informed consent, in accordance with institutional bioethical guidelines and the Declaration of Helsinki. Mouse brain tissue (C57BL/6) was purchased from Innovative Research, Inc. (Novi, MI, USA). Human embryonic kidney (HEK-293) cell line purchased from American Type Culture Collection (ATCC, Rockville, MD, USA).

### Synthesis

PEEK-sil tubing was pretreated following the procedure reported in our previous study.^22^ For the silanization step, the tubing was filled with a 50:50 (v/v) solution of 3-(trimethoxysilyl)­propyl methacrylate in methanol, sealed with end-caps, and heated at 90 °C for 12 h. After treatment, the tubing was thoroughly rinsed with methanol and dried under a nitrogen stream. Subsequently, the surface-modified PEEK-sil tubing was functionalized via a thiol–ene click reaction. The prepolymerization mixture consisted of *N*,*N*′-methylenebis­(acrylamide) (0.10 mmol, 15 mg), pentaerythritol tetrakis­(3-mercaptopropionate) (0.05 mmol, 24 mg), DGDE (0.47 mmol, 95 mg), PEG (0.68 mmol, 136 mg), and AIBN (0.03 mmol, 5 mg) in 1 mL of ethanol. The mixture was sonicated and then homogenized by vortex mixing for 5 min. The resulting solution was introduced into the pretreated PEEK-sil tubing, which was subsequently sealed and maintained at 90 °C overnight to allow polymerization. After synthesis, the functionalized PEEK-sil column was washed three times with a methanol/acetic acid mixture (9:1, v/v), followed by a methanol rinse to remove residual acetic acid. The column was finally dried under a nitrogen flow. The synthesized capillary column was designated as TE-PLOT, referring to a thiol–ene porous layer open tubular stationary phase. Five batches of TE-PLOT columns were prepared and tested for the reproducibility of selected standards; parallel online enrichment experiments were performed, and their performances were compared.

### Characterization of the TE-PLOT Column

Vertical cross sections of the TE-PLOT columns were characterized by scanning electron microscopy (SEM) for morphological analysis using a Quanta 200 microscope (FEI Company) equipped with an X-ray energy-dispersive spectroscopy (X-EDS) detector (Oxford INCA-350, Bruker). Elemental microanalysis was performed by energy-dispersive X-ray spectroscopy (X-EDS) using a Nova NanoSEM 450 (FEI Company) coupled with a Quantax 200 system (Bruker). Prior to SEM analysis, the samples were sputter-coated with a thin chromium layer to enhance conductivity. The SEM analyses were carried out on two different TE-PLOT columns to assess column-to-column reproducibility. Spectroscopic characterization was carried out by Fourier-transform infrared (FTIR) microspectroscopy using a Lumos II IR microscope (Bruker). The polymeric material was synthesized in bulk under identical polymerization conditions to those used for capillary preparation, followed by extensive washing with methanol and drying prior to FTIR analysis. FTIR spectra were collected in the 4000–500 cm^–1^ range with a spectral resolution of 8 cm^–1^. Forty scans were acquired for each spectrum and averaged before Fourier transformation. Background spectra were recorded and subtracted for each measurement. Adsorption experiments were performed to investigate the thermodynamics of the adsorption process using the Langmuir, Freundlich, and Scatchard models, as described in the Supporting Information and in accordance with previously reported literature.^23^ The hydrodynamic properties of the PLOT capillary were evaluated through pressure–flow measurements. A water/acetonitrile (50:50, v/v) mixture was pumped through the capillary at different flow rates, and the corresponding pressure drop was recorded. The effective hydraulic radius (*R*_c_) of the residual open channel was calculated using the Hagen–Poiseuille eq 1:

1 $$\Delta P=\frac{\left(8\mu LQ\right)}{\pi {R_{c}}^{4}}$$

where Δ*P* is the measured pressure drop (Pa), μ is the dynamic viscosity of the mobile phase (0.97 × 10^–3^ Pa·s for water/acetonitrile 50:50 at 20 °C), *L* is the capillary length (m), and *Q* is the volumetric flow rate (m^3^ s^–1^).

The hydraulic permeability (*K*_0_) was calculated according to eq 2:

2 $$K_{0}=\frac{{R_{c}}^{2}}{8}$$

which is commonly used for open-tubular and monolithic capillary systems.^24^

The flow porosity (ε_f_), corresponding to the fraction of the original capillary cross-section available for convective flow, was estimated as (eq 3):

3 $$\varepsilon_{f}={\left(\frac{R_{c}}{R_{0}}\right)}^{2}$$

where *R*_0_ is the internal radius of the untreated capillary.^25^ The geometrical surface area directly accessible to the mobile phase was estimated using eq 4:

4 $$A_{\mathrm{geom}}=2\pi R_{c}L$$

### Online Sample Preparation Program

The online sample preparation protocol was optimized using a standard mixture at 20 ng mL^–1^ containing 5-HT, DA, GLU, GSH, and GABA, with an injection volume of 0.1 mL, in order to investigate the key variables affecting the IT-SPME procedure based on TE-PLOT columns. The evaluated parameters included: loading solvent (water, acetonitrile, methanol); percentage of acetonitrile in water (0–90%); NH_4_OH content (0.01–0.1%); aspiration flow rate (0.03–1.80 mL min^–1^); dispensing flow rate (0.03–1.80 mL min^–1^); and elution flow rate (0.03–0.15 mL min^–1^). Instrumental conditions were adjusted using a user-defined program implemented within the method editor. Direct injection without the TE-PLOT column was employed as a comparative control. All experiments were performed in triplicate under each tested condition. The TE-PLOT column was integrated into an Ultimate 3000 UHPLC system (Thermo Fisher Scientific, Milan, Italy), equipped with a degasser, pump, thermostated column compartment, a syringe valve for automated needle and loop washing, an autosampler fitted with a 100 μL injection loop, and a programmable six-port Rheodyne switching valve (Figure 1). For column activation and conditioning, the valve was set to the loading position (1–6). Subsequently, 100 μL of Washinh Solution (H_2_O containing 0.05% NH_4_OH) was aspirated and passed through the SPME device to waste. This step was repeated twice. Then, 100 μL of the sample was loaded and discarded. After two additional washing steps with Washinh Solution, the valve was switched to the injection position (1–2), allowing the mobile phase to flow through the TE-PLOT column and subsequently into the analytical chromatographic column for analyte elution. A nonfunctionalized PEEK-sil tubing was also tested instead of the TE-PLOT columns for comparison.

**Figure 1:**
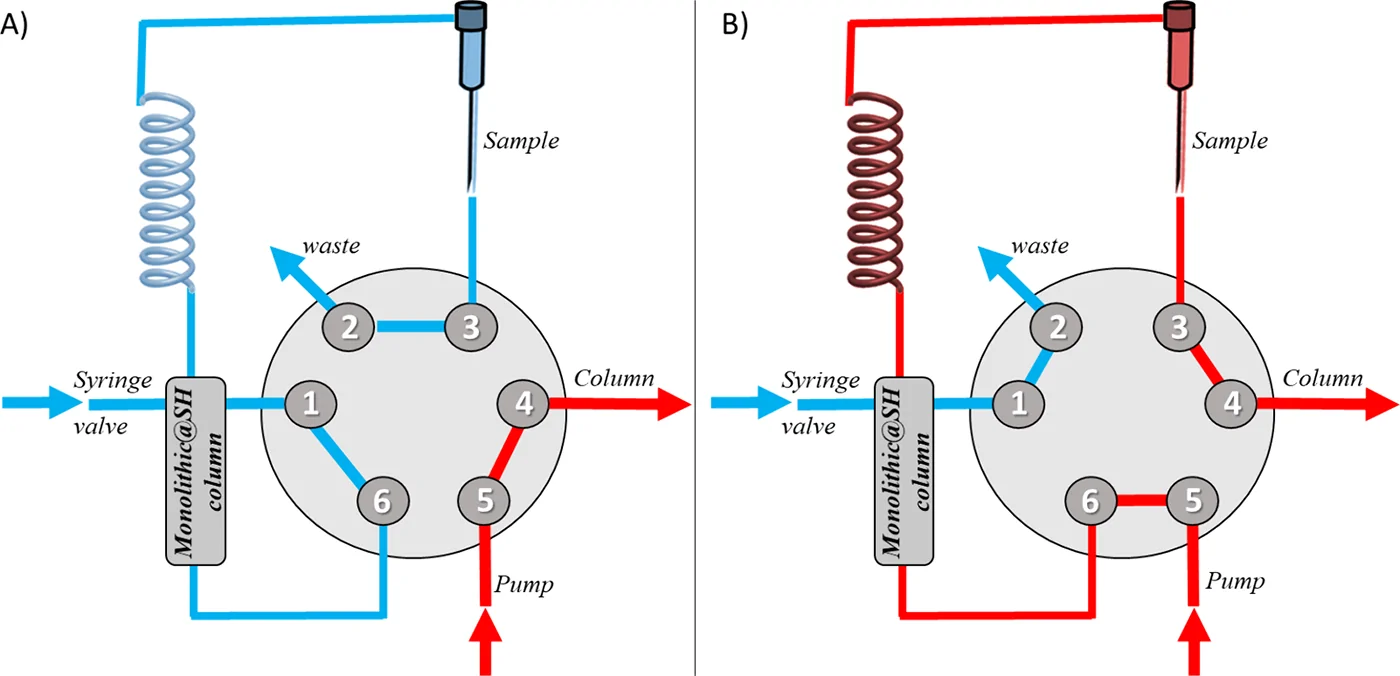
Online sample preparation program. (A) Valve in position 1–6: conditioning, sample loading, and washing steps. (B) Valve in position 1–2: elution step.

#### Stability, Reusability, and Storage Tests

The stability of the TE-PLOT column was assessed under different pH conditions: 0.05% NH_4_OH in water/acetonitrile 50/50 v/v (loading pH ≈ 10) and 0.1% formic acid (elution pH ≈ 3). For these experiments, the columns were continuously flushed with the corresponding solution for 2 h at a flow rate of 0.15 mL min^–1^, after which their extraction performance was evaluated. Column performance was also assessed after repeated use (1–250 extraction cycles) and following storage for up to 12 months. After each use, the columns were rinsed with water followed by methanol, sealed, and stored at 21 °C in the dark until subsequent use. Performance evaluation was carried out using a quality control (QC) sample containing all target analytes at 20 ng mL^–1^ [5-hydroxytryptamine (5-HT), dopamine (DA), glutamate (GLU), glutathione (GSH), and γ-aminobutyric acid (GABA)]. Mean analyte recoveries were determined under each experimental condition and used as the primary metric to assess column performance.

### Preparation of Samples

Blood samples were allowed to clot for 30 min and were then centrifuged at 2000 × g for 10 min at 4 °C. Plasma was collected, aliquoted, and stored at −80 °C until analysis. Prior to each enrichment step, plasma aliquots were thawed at 4 °C and centrifuged at 12000 × g for 10 min to remove insoluble debris. For protein precipitation, 200 μL of plasma was diluted 1:1 (v/v) with acetonitrile containing 0.2 mmol L^–1^ EDTA and 1 μmol L^–1^ ascorbate. The mixture was vortexed briefly and centrifuged at 12000 × g for 10 min at 4 °C. The clear supernatant was transferred to a clean vial for online in-tube SPME and UHPLC–MS/MS analysis.

Cells were maintained in Dulbecco Modified Eagle Medium (DMEM) (Gibco Life Technologies) supplemented with 2 mmol L^–1^ l-glutamine, 100 IU/mL penicillin/streptomycin, and Fetal Bovine Serum (FBS) (Gibco Life Technologies) 10% and under a humidified atmosphere at 37 °C and 5% CO_2_. Cells were detached and collected at a density of 3 × 10^6^ cells in 5 mL round-bottom polystyrene Falcon tubes. After centrifugation at 300 × g for 3 min at 4 °C, the cell pellet was resuspended in 200 μL of Phosphate buffer (PBS, pH 7.4, 0,01 mol L^–1^) containing 0.2 mmol L^–1^ EDTA and 1 μmol L^–1^ ascorbate and treated with 200 μL of acetonitrile (1:1, v/v). The mixture was vortexed and centrifuged at 12000 × g for 10 min at 4 °C. The supernatant was transferred to a clean vial for online in-tube SPME and UHPLC-MS/MS analysis. Frozen mouse brain samples stored at −80 °C were weighed and processed on ice. Approximately 50–60 mg of tissue were homogenized in 200 μL of ice-cold PBS buffer. using a bead-based homogenizer. Protein precipitation was performed by adding 800 μL of ice-cold acetonitrile (1:1, v/v). The mixture was vortexed and centrifuged at 12000 × g for 10 min at 4 °C. The clear supernatant was collected and transferred to a clean vial for online in-tube SPME and UHPLC-MS/MS analysis.

### UHPLC-MS/MS analysis

UHPLC-MS/MS analyses were performed using an Ultimate 3000 UHPLC system equipped with a binary pump (Thermo Fisher Scientific, Bremen, Germany) interfaced to a TSQ Vantage EMR triple quadrupole mass spectrometer (Thermo Fisher Scientific, Bremen, Germany) through a heated electrospray ionization (HESI) source. Instrument operation, data acquisition, and processing were managed with Xcalibur software (version 2.2, Thermo Fisher Scientific). Chromatographic separation was carried out on a Luna Polar Pesticide analytical column (2.1 × 100 mm, 3 μm particle size; Phenomenex) coupled to a matching guard column. The column temperature was maintained at 55 °C, and the mobile phase flow rate was set at 0.15 mL min^–1^. The mobile phases consisted of water (A) and acetonitrile (B), each containing 0.1% (v/v) formic acid. The gradient elution program was as 0–4 min, 99% B (isocratic); 4–10 min, linear decrease to 40% B; 10–11 min, further decrease to 30% B; 11–15 min, isocratic at 30% B; 15–16 min, linear increase back to 99% B; 16–21 min, re-equilibration at 99% B. The HESI source was operated in positive ion mode with a spray voltage of +4.0 kV. The vaporizer and ion transfer tube temperatures were set at 300 and 275 °C, respectively. Sheath gas, auxiliary gas, and sweep gas were set to 45, 4, and 0 arbitrary units, respectively. Mass calibration and resolution tuning of the quadrupoles were performed automatically on a monthly basis using the manufacturer’s calibration solution to maintain mass accuracy and instrument performance. For method optimization, standard solutions (10 μg L^–1^) were prepared and directly infused at 10 μL min^–1^. Two selected reaction monitoring (SRM) transitions were monitored for each analyte: the most intense transition was used for quantification, and a second transition was employed for confirmation. S-lens RF levels and collision energies were optimized individually for each SRM transition (see Table 1).

**Table 1:** Molecular Formulas, Precursor and Product Ions, And Optimized MS Parameters^a^

compound	chemical formula	precursor ion [M + H]^+^	product ions *m*/*z*	collision energy (CE)	S-lens voltage (Hz)
5-HT	C_10_H_12_N_2_O	177	**160.2**	11	54
			89.3	50	
DA	C_8_H_11_NO_2_	154	**91.3**	21	60
			119.2	16	
GLU	C_5_H_9_NO_4_	148	**84.3**	15	59
			56.4	26	
GSH	C_10_H_17_N_3_O_6_S	308	**162.1**	15	94
			76.3	21	
GABA	C_4_H_9_NO_2_	104	**45.5**	20	51
			69.4	15	

a Quantifier transitions are indicated in bold.

### Method Validation

The method was validated in accordance with FDA bioanalytical guidelines (M10 Bioanalytical Method Validation) (https://www.fda.gov/regulatory-information/search-fda-guidance-documents/m10-bioanalytical-method-validation-and-studysample-analysis, last accessed on 23/05/2024), and following previously reported validation strategies for LC-MS/MS assays.^22^ Assessed parameters included recovery (RE), matrix effect (ME), linear dynamic range, precision, and limits of detection and quantification (LOD and LOQ). Recovery was determined at three fortification levels by comparing the peak areas of samples spiked with pure standards before analysis, subtracting the endogenous contribution from the areas of pure standards injected directly into the instrument without the in-tube microextraction process (eq 5), where C_1_ is the fortified sample placed inside the autosampler for the in-tube SPME process; C_0_ is the nonfortified sample extracted under the same conditions; C_s_ is the solvent fortified with pure standards and injected directly without the in-tube SPME process.

5 $$\mathrm{RE}=\frac{\mathrm{Area}C_{1}-\mathrm{Area}C_{0}}{\mathrm{Area}C_{s}}\times 100$$

Matrix effects were assessed by comparing the responses of the analytes in the matrix with those obtained from standards prepared in solvent under equivalent instrumental conditions (eq 6), where C_2_ is the solvent fortified with pure standards placed in the autosampler for the in-tube SPME.

6 $$\mathrm{ME}=\frac{\mathrm{Area}C_{1}-\mathrm{Area}C_{0}}{\mathrm{Area}C_{2}}\times 100$$

Calibration curves were constructed in solvent over the validated concentration range, and all measurements were performed in triplicate. Intraday precision was calculated as the RSD from six replicate analyses performed within a single day, whereas interday precision was assessed over five consecutive days using five independently prepared TE-PLOT columns (*n* = 5), each analyzed in triplicate. The LOD was experimentally established as the lowest concentration allowing a signal-to-noise ratio ≥ 3 with MRM acquisition,^26^ considering the inherently low baseline noise of tandem MS measurements. Since true blank biological matrices were not available due to the endogenous presence of neurotransmitters, the estimated LOD values were further verified by serial dilution experiments of standard solutions until the lowest detectable concentration was identified. The LOQ was defined as the lowest concentration within the validated linear range.

## Results and Discussion

### Column Preparation and Characterization

The thiol–ene porous layer stationary phase column (TE-PLOT) was designed to combine the high permeability typical of open-tubular structures with controlled surface functionality, two key parameters for efficient in-tube SPME applications. To achieve this, the molar ratio between thiol and alkene monomers was carefully optimized to ensure the formation of a mechanically stable and porous network. The surface activation of the PEEK-sil tubing enabled covalent anchoring of the monolith, preventing detachment under flow conditions and ensuring reproducible performance during repeated analytical cycles. The resulting material was designed to provide a high density of accessible interaction sites while maintaining low backpressure, a critical requirement for online coupling with UHPLC systems.

The preparation procedure is schematically illustrated in Figure 2. The inner silica surface of the PEEK-sil tubing was first activated by sequential basic and acidic treatments, as previously described.^23^ Subsequently, the free silanols were functionalized with 3-(trimethoxysilyl)­propyl methacrylate; this functionalization introduces double bonds on the surface for the subsequent thiol–ene click reaction. The polymerization mixture consisted of the alkene-containing monomer *N*,*N*′-methylenebis­(acrylamide) and the thiol-containing monomer pentaerythritol tetrakis­(3-mercaptopropionate) at a fixed molar ratio of 2:1. Polymerization was initiated by free radicals generated from AIBN.

**Figure 2:**
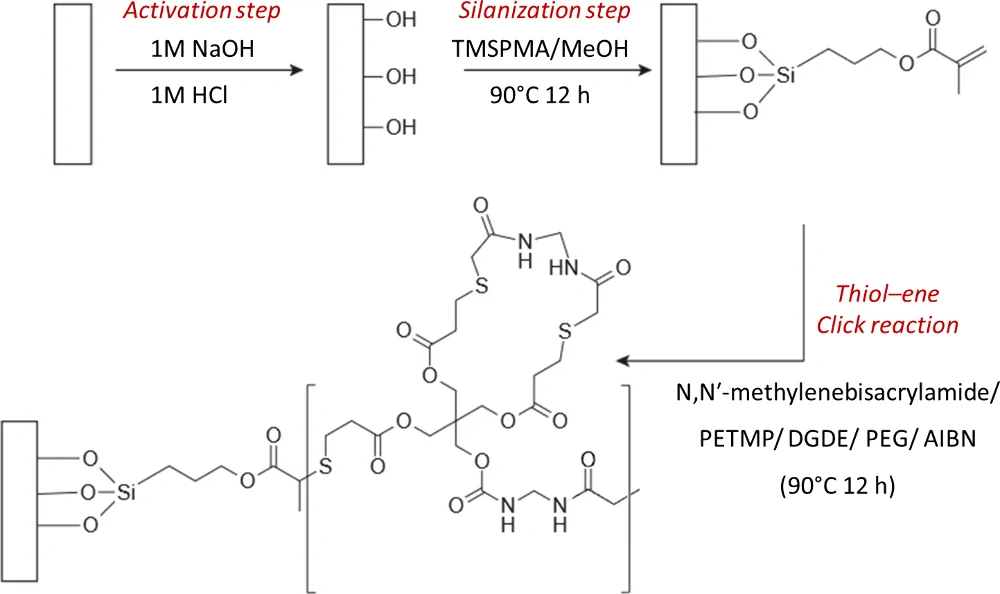
Schematic of the synthesis of the TE-PLOT column for SPME, activation of silanol groups, functionalization with 3-(trimethoxysilyl)­propyl methacrylate, and thiol–ene click reaction for polymerization.

The thiol–ene reactions proceed through a typical radical “click” mechanism, highly efficient and characterized by high yields, as widely described in the literature;^27^ this reaction represents an efficient step-growth radical reaction with minimal formation of byproducts. In addition to the reactive monomers, diethylene glycol dibutyl ether (DGDE) and polyethylene glycol (PEG200) were incorporated as porogenic components to regulate phase separation and control the morphology of the porous polymer layer. Although the high permeability of the column primarily derives from its open-tubular architecture, the composition of the porogenic mixture plays a critical role in ensuring complete monomer solubility before polymerization and in governing the phase separation process, which ultimately determines the porosity and specific surface area of the resulting layer.^20^ In fact, PEG can act as a flexible segment in the polymer network,^28^ controlling cross-linking density and mechanical properties.^29^ DGDE acts as a strong, high-boiling ether solvent in these systems, allowing operation at elevated temperatures.^30^

### TE-PLOT Column Characterization

The morphology and composition of the synthesized TE-PLOT stationary phase were investigated by scanning electron microscopy (SEM), energy-dispersive X-ray spectroscopy (EDX), Fourier transform infrared spectroscopy (FTIR), and hydrodynamic measurements. Three independently prepared columns were characterized to assess the reproducibility of the synthesis procedures.

Representative SEM images are shown in Figure 3. Cross-sectional observations revealed the typical architecture of a PLOT column, consisting of an external PEEK housing, an inner fused-silica capillary, and a continuous polymer coating deposited on the inner wall. The coating exhibited homogeneous morphology along the capillary wall, and no evidence of detachment or structural collapse was observed after repeated use. In particular, Figure 3B shows that the coating of the material is present exclusively on the inner surface of the capillary, forming a continuous thiol–ene coating firmly anchored to the inner wall of the capillary. In Figure 3A,B, the different layers constituting the column are clearly distinguishable: the outer PEEK layer, which provides mechanical strength; the inner silica capillary, which is readily functionalizable; and finally, porous-layer stationary phase deposited on the internal surface. Low-magnification SEM images (Figure 3B) clearly show the capillary cross-section. The stationary-phase layer appears dense and compact, with no diffuse macropores within the solid matrix, while signs of mechanical processing associated with capillary cleavage are visible (Figure S1). The structure is characterized by a single central macroscopic hollow channel, typical of TE-PLOT configurations. At higher magnification (Figure 3C), however, the surface exhibits a characteristic microglobular rough texture, typical of porous thiol–ene polymer networks synthesized in the presence of porogenic solvents (DGDE and PEG200), which promote phase separation during polymerization. Crystalline fragments and micrometer-scale surface asperities are also visible, whereas no interconnected pore network is observed within the bulk of the coating.

**Figure 3:**
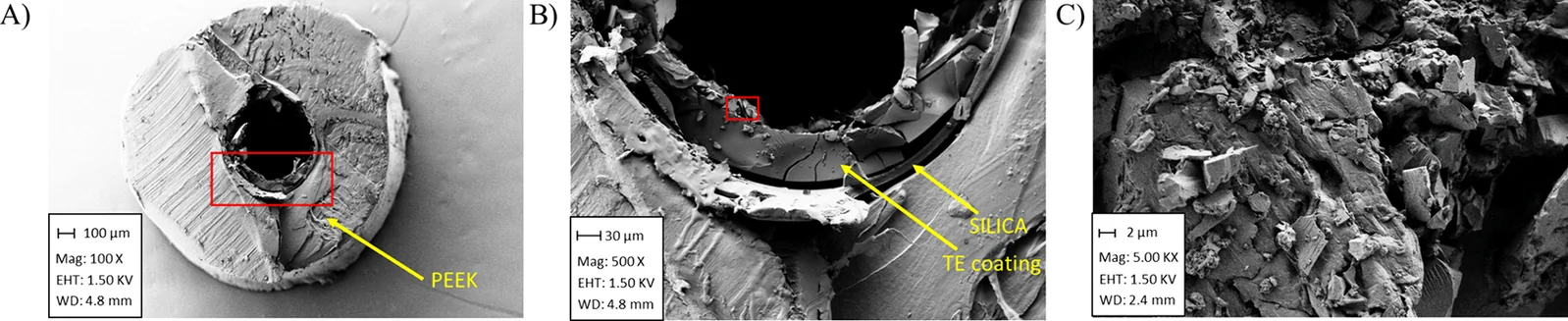
SEM characterization of thiol–ene porous layer capillary column cross-section. (A) Overview of the column cross-section showing the external PEEK housing. (B) Higher-magnification image highlighting the concentric layers identified the fused-silica capillary, and the coated inner region was confirmed by EDX analysis. (C) Detailed view of the coating deposited on the inner surface of the fused-silica capillary, showing its morphology and thickness. Magnification values are reported in each micrograph.

SEM analyses performed on three independently prepared TE-PLOT columns showed the same layered architecture and comparable coating morphology, demonstrating the good reproducibility of the synthesis procedure. The hydrodynamic and structural parameters were not evaluated using conventional porosity analysis software but were instead directly derived from the geometric dimensions of the channel using classical methods. Obtaining perfectly intact cross sections proved challenging because mechanical sectioning frequently induced local fracture or deformation of the polymer layer (Figure S1); nevertheless, the preserved regions were suitable for morphological and compositional characterization. The area selected for EDX is shown in Figure 3C.

The chemical composition of the coating was further investigated by EDX mapping (Figure 4). The different images represent the spatial distribution of the main elements present in the porous layer of the TE-PLOT column; the EDS microanalysis of the TE-PLOT in Figure S2 shows the presence of carbon (53.28 wt %), oxygen (27.67 wt %), sulfur (12.20 wt %), and silicon (6.85 wt %). Sulfur was localized within the polymer coating, supporting the successful incorporation of sulfur-containing functionalities derived from the thiol–ene polymerization (Figure 4A). This result is consistent with the successful functionalization via thiol–ene click chemistry, which leads to the introduction of sulfur-containing groups on the coating layer surface.

**Figure 4:**
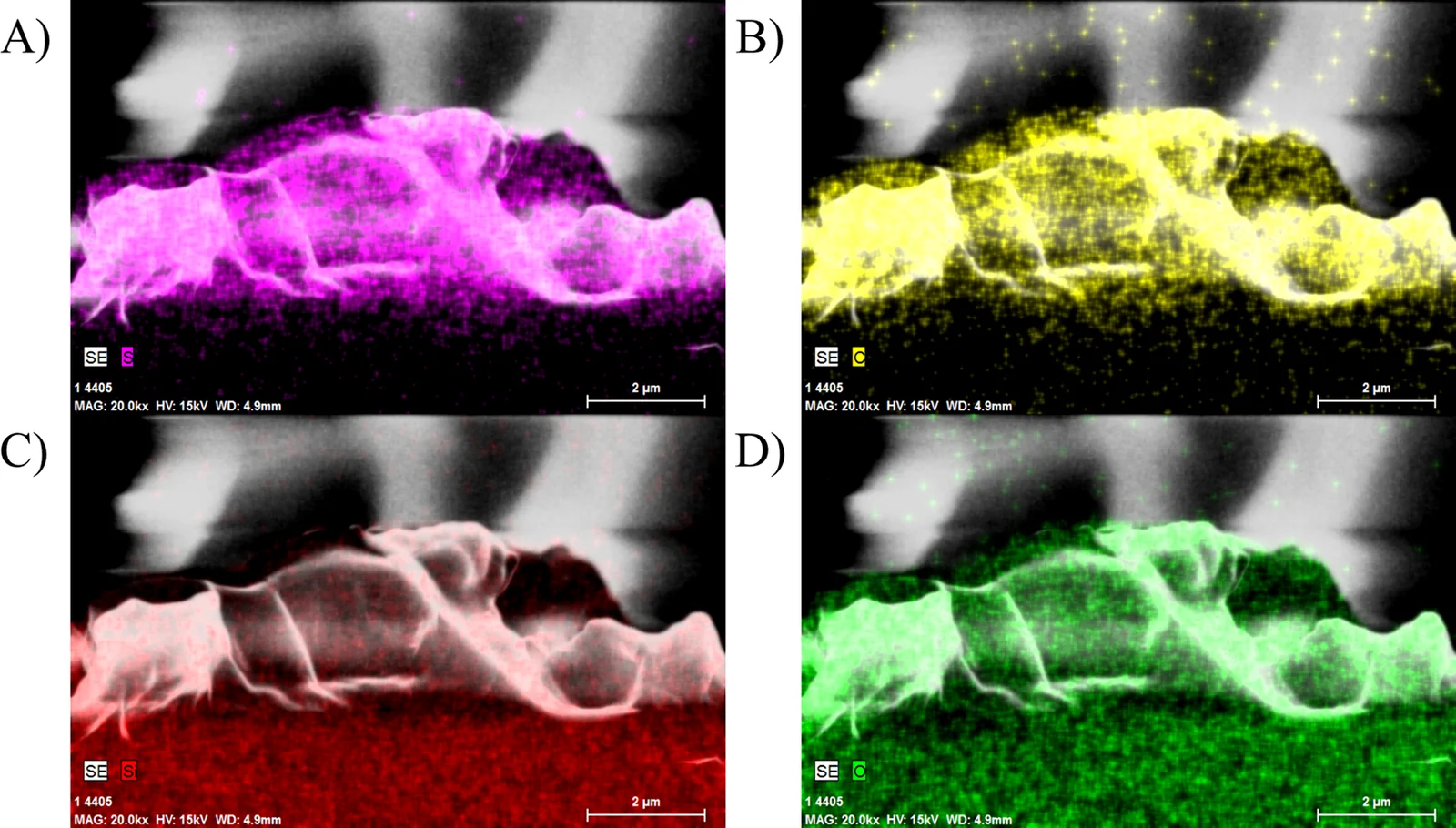
Energy-dispersive X-ray spectroscopy (EDX) elemental maps of the analyzed section of the functionalized inner layer. (A) Sulfur distribution (pink), (B) carbon distribution (yellow), (C) silicon distribution (red), and (D) oxygen distribution (green).

Carbon was uniformly distributed throughout the coating, consistent with the organic polymer matrix (Figure 4B), whereas silicon was predominantly associated with the fused silica support but was also detected at the coating interface owing to surface silanization (Figure 4C). Oxygen is distributed both in the region associated with the silica support and within the functionalized porous layer (Figure 4D). This is consistent with the composition of the system as oxygen is present both in the silica structure (SiO_2_) and in the functional groups introduced into the porous coating during the synthesis and chemical modification steps.

Additional structural information was obtained by FTIR spectroscopy (Figure 5). In the region around 1700–1750 cm^–1^, a very intense absorption band is observed, attributable to C=O stretching (carbonyl). Additionally, multiple strong bands in the 1000–1300 cm^–1^ range are assigned to C–O stretching and C–C/C–O vibrational modes, characteristic of ether functionalities. Finally, a broad and weak band between 3300–3500 cm^–1^ is attributed to O–H stretching, likely arising from water adsorbed by the material, which may obscure any potential N–H contribution that could be overlapped and not clearly distinguishable. Comparison of the spectrum of the synthesized material with that of the individual monomers revealed significant differences. In particular, the disappearance of S–H stretching bands in the ∼2550–2600 cm^–1^ and 2800–3000 cm^–1^ region, clearly visible in the monomer pentaerythritol tetrakis­(3-mercaptopropionate) (Figure S3A) was observed. The monomer *N*,*N*′-methylenebis­(acrylamide) (Figure S3B) also exhibits a distinct spectrum that does not overlap with that of the synthesized material. Its sharp band at ∼3300 cm^–1^ suggests N–H stretching, and the presence of a single peak is indicative of a secondary amine. Notably, this is the same spectral region that, in the synthesized material (Figure 5), is difficult to interpret due to the presence of adsorbed water. Furthermore, the fingerprint region of the monomer spectrum indicates the presence of multiple double bonds, which are absent in the synthesized material as a consequence of thiol–ene click polymerization.

**Figure 5:**
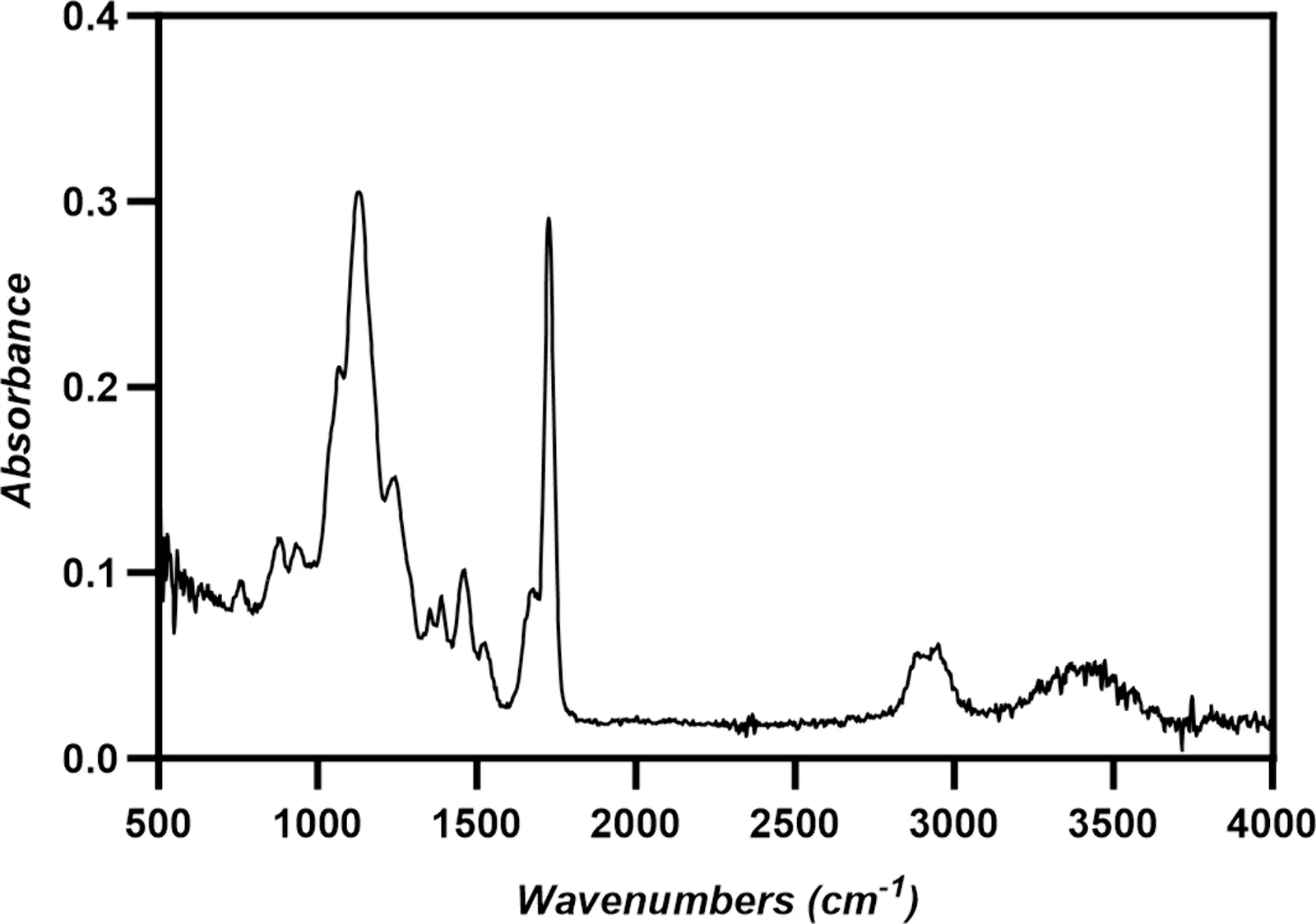
IR spectrum of the synthesized material inside the TE-PLOT column; polymerization was carried out in bulk to study the structure.

The hydrodynamic behavior of the PLOT capillary was investigated. Under optimized operating conditions (water/acetonitrile 50:50, v/v; flow rate 0.15 mL min^–1^), a pressure drop of 5.45 bar was measured across the 15 cm capillary. This represents an optimal pressure level, making the device suitable for in-tube SPME, as higher pressures would not have allowed its implementation in an autosampler system. Application of the Hagen–Poiseuille model (eq 1) yielded an effective hydraulic radius (Rc) of 36.1 μm, corresponding to an equivalent hydraulic diameter of 72.2 μm. This value is smaller than the physical lumen radius estimated from SEM observations (∼50 μm), indicating that the hydrodynamic behavior of the TE-PLOT capillary cannot be described solely by solvent transport through the central open channel. Instead, the mobile phase partially permeates the porous thiol–ene polymer layer, contributing to the overall convective transport and increasing the effective flow resistance compared with an ideal smooth open tubular capillary. This interpretation is consistent with the microglobular surface morphology observed by SEM, where the rough and porous architecture provides additional pathways for mass transfer beyond the central void. Thus, the observed pressure drop reflects the combined contribution of flow through the central channel and convective transport within the porous stationary phase. The hydraulic permeability was calculated as 1.63 × 10^–10^ m^2^ (eq 2), indicating efficient convective transport with limited hydraulic resistance. This value falls within the range typically reported for open-tubular and porous-layer capillary systems.^24^ The flow porosity (ε_f_), defined as the fraction of the capillary cross-section available for mobile-phase transport, was estimated to be 1.86% (eq 3). This result indicates that most of the capillary volume is occupied by the deposited thiol–ene stationary phase while still preserving a continuous channel for solvent flow. The geometrical surface area accessible to the mobile phase was estimated as 0.340 cm^2^ (eq 4). The porous nature of the stationary phase is further supported by adsorption measurements. The high extraction capacity observed for representative analytes (*Q* ≈ 500 ng cm^–1^ for 5-HT and GSH) indicates the presence of a highly accessible interaction surface generated by the porous microarchitecture, which cannot be provided by a conventional nonporous smooth coating. Therefore, the combination of hydrodynamic data, SEM characterization, and adsorption performance provides complementary evidence of the porous-layer architecture of the synthesized TE-PLOT phase. Considering the intrinsic limitations associated with the characterization of capillary porous materials, as highlighted by Svec,^31^ who discussed the challenges of applying bulk techniques such as BET analysis to these systems, the combined evaluation of SEM imaging, hydrodynamic parameters, and adsorption performance represents a more suitable approach for confirming the porous nature of the coating. The combination of low backpressure, adequate permeability, porous-layer morphology, and preserved open-channel geometry demonstrates that the synthesized material behaves as a PLOT stationary phase suitable for online in-tube SPME applications.

### Optimization Procedure

#### Optimization of the Online Loading Method

The influence of loading solvent composition on analyte retention was systematically evaluated to elucidate the interaction mechanisms governing adsorption. Water, acetonitrile, and methanol were evaluated, as shown in Figure 6A.

**Figure 6:**
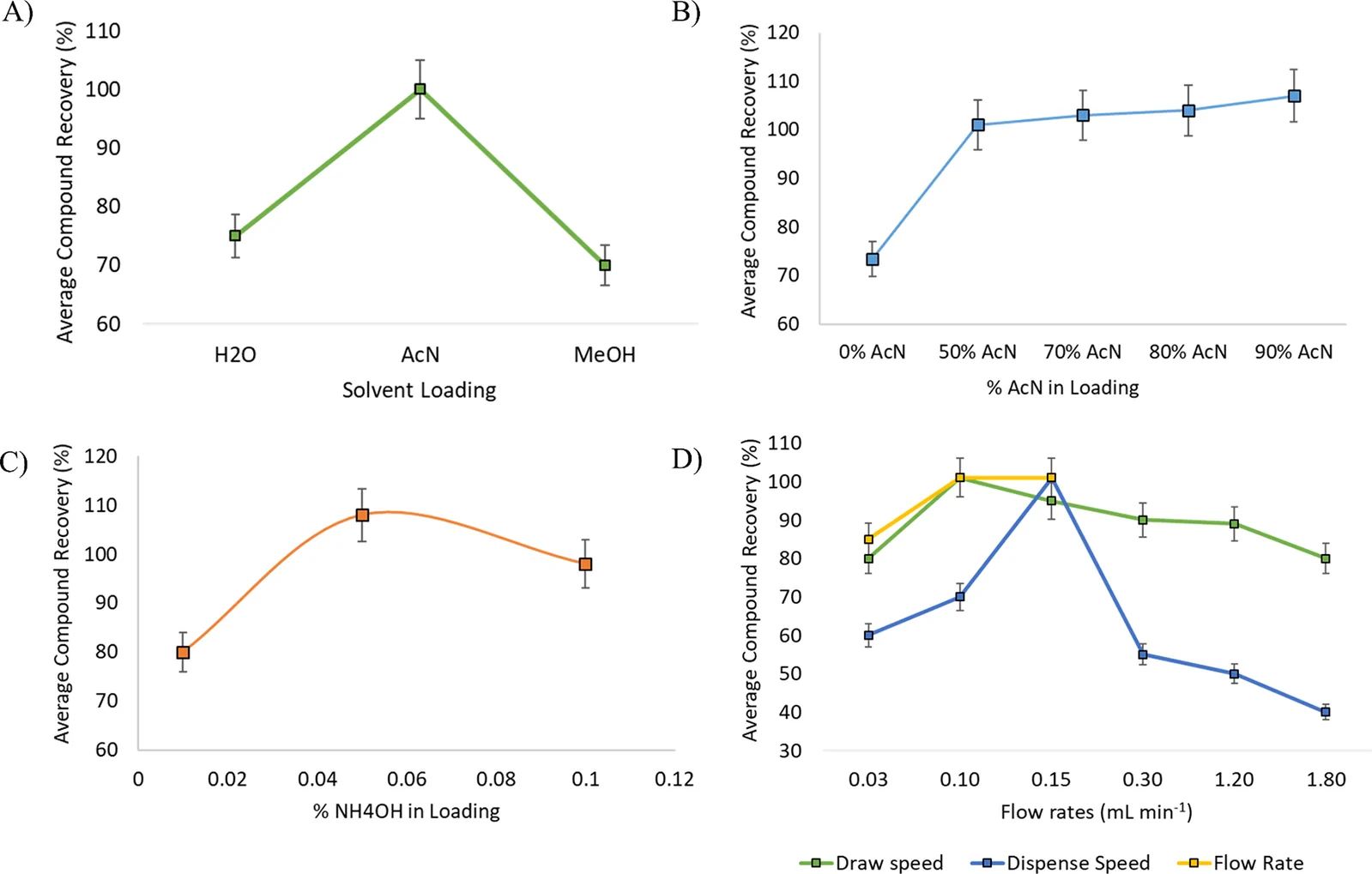
Optimization of loading and elution conditions on TE-PLOT column: (A) effect of loading solvent (water, acetonitrile, methanol); (B) influence of acetonitrile percentage in water; (C) effect of NH_4_OH concentration; (D) effect of flow rate on adsorption performance.

Results are reported as averages of the selected standards; thus, loading and recovery percentages correspond to mean values calculated across all standards included in the study. The concentration investigated in these studies was 20 ng mL^–1^ for all standards. Acetonitrile provided the highest adsorption efficiency (>90%), indicating that reduced solvation of polar analytes promotes their interaction with the stationary phase, likely due to the presence of multiple functional groups on the porous layer stationary phase, which enables secondary interactions, including hydrogen bonding and dipole–dipole interactions, which further stabilize analyte adsorption. This suggests that adsorption derives from the combined contribution of different interaction mechanisms rather than a single dominant process. Overall, analyte retention is best described as a mixed-mode HILIC-like mechanism, dominated by hydrophilic interactions under the optimized loading conditions, with additional contributions from hydrogen bonding, dipole–dipole, and electrostatic interactions depending on the physicochemical properties of the analytes. Given that real samples were prepared in aqueous solution, the acetonitrile content in water was optimized, also enabling protein precipitation (Figure 6B). Optimization of acetonitrile content revealed a maximum adsorption at 50% (v/v). These results are consistent with a HILIC-like retention mechanism, in which highly polar analytes are expected to partition into a water-enriched layer associated with the stationary phase.^32,33^ Although the predominant extraction mechanism is HILIC-like, the organic thiol–ene polymer may also provide secondary analyte-dependent interactions. In particular, aromatic compounds can establish secondary hydrophobic interactions with the polymeric backbone, contributing to the overall retention without changing the predominant HILIC-like behavior of the extraction phase. In addition to partitioning, hydrogen bonding and dipole–dipole interactions between polar functional groups on the stationary phase and the analytes are also likely to contribute to retention.

The addition of NH_4_OH (0.02–0.12%) in acetonitrile was also evaluated (Figure 6C), demonstrating a crucial role in modulating analyte retention through the regulation of their ionization state. Under mildly basic conditions, NH_4_OH regulates the ionization state of analyte functional groups, modulating the balance among hydrophilic partitioning, hydrogen bonding, dipole–dipole interactions, and electrostatic contributions. This balance between analyte charge state and interaction strength is essential to achieve efficient retention while preserving effective desorption during the elution phase.

SEM images confirm that the use of 0.05% NH_4_OH does not cause any corrosion or structural collapse of the silica, which retains its morphology even after use. Furthermore, surface functionalization via silanization, followed by click chemistry, acts as a protective shield for the silica core.

The user-defined method allowed independent control of flow rates and valve movements during injection, including predefined aspiration and dispensing delays. Elution flow rate was first investigated at 0.03 and 0.15 mL min^–1^ (Figure 6D), yielding near-quantitative results; higher flow rates were not feasible due to excessive backpressure leading to detachment of the TE-PLOT column. Therefore, 0.15 mL min^–1^ was selected to ensure faster analysis and improved separation on the analytical column. The dispensing rate (0.03–1.8 mL min^–1^) significantly affected performance, with optimal results at 0.15 mL min^–1^, whereas both lower and higher values resulted in reduced analyte binding (<60%). This behavior highlights the key role of mass transfer kinetics in the extraction process: at higher flow rates, reduced residence time limits analyte–surface interactions, while excessively low flow rates may lead to diffusion-limited transport. In contrast, the aspiration rate (0.03–1.8 mL min^–1^) showed negligible influence on performance, and 0.1 mL min^–1^ was selected as the optimal condition, indicating that adsorption is primarily governed during the active contact phase between the sample and the stationary phase.

Following adsorption on the TE-PLOT column, elution was performed under HILIC conditions using acetonitrile and water, both containing 0.1% formic acid. This choice is consistent with the retention mechanism described above. In HILIC systems, decreasing the organic solvent content weakens the partitioning of analytes into the water-enriched layer associated with the stationary phase, thereby promoting their transfer back to the mobile phase. The introduction of formic acid further facilitates desorption by modulating the ionization state of both analytes and functional groups on the stationary phase. Under acidic conditions, protonation of amine-containing analytes reduces their ability to participate in hydrogen bonding and weakens polar interactions with carbonyl and heteroatom sites. At the same time, competition with the mobile phase for hydrogen-bonding interactions increases, further destabilizing the interaction network responsible for retention. As a result, the combined effect of reduced partitioning and weakened specific interactions enables the efficient and controlled elution of highly polar compounds. Thus, the elution process remains consistent with a HILIC-based mechanism, where decreasing the organic solvent content progressively disrupts the hydrophilic partitioning process responsible for analyte retention, while formic acid assists desorption by weakening specific polar interactions rather than indicating a predominant reversed-phase retention mechanism. To ensure that the absorption was attributable to the material synthesized inside the capillary via the click reaction, a nonfunctionalized PEEK-sil tubing was also tested. As shown in Figure S5, the analytes were not retained by the bare silica, confirming that the absorption occurs specifically due to the functional groups introduced within the capillary.

#### Column Stability, Reusability, and Storage Performance

The TE-PLOT column exhibited excellent stability and reproducibility under all tested conditions. Mean recoveries for all investigated analytes remained close to 100% across the evaluated pH conditions (0.05% NH_4_OH loading solution and 0.1% formic acid elution solution), demonstrating that neither the loading nor the elution step adversely affected analyte retention or desorption efficiency (Figure S6-A). Similarly, repeated column use up to 250 extraction cycles resulted in no appreciable loss of performance, with recoveries remaining within the experimental variability observed for freshly prepared columns (Figure S6-B). Long-term storage studies further confirmed the robustness of the sorbent material. Columns stored for up to 12 months at room temperature (21 °C) in the dark after washing with water and methanol showed no significant decrease in analyte recovery (Figure S6-C). The absence of systematic trends in recovery values over time indicates that the extraction phase is chemically stable and resistant to degradation under the adopted storage conditions. Notably, the recoveries obtained for chemically diverse analytes, including the biogenic amines 5-hydroxytryptamine and dopamine, the amino acid neurotransmitters glutamate and γ-aminobutyric acid, and the tripeptide glutathione, were consistently maintained throughout the study. These results highlight the robustness of the TE-PLOT platform and support its suitability for routine analytical applications requiring repeated use and long-term storage without compromising extraction efficiency.

#### Adsorption Studies

After establishing the optimal loading solvent, adsorption studies were performed to further elucidate the interaction mechanisms governing analyte retention on the TE-PLOT column. The experimental data were fitted using Langmuir, Freundlich, and Scatchard isotherm models (Figures S7–S9; Table 2). Two representative analytes with markedly different polarity were selected, namely 5-HT (log P: 0.2) and GSH (log P: 4.5), to probe the influence of physicochemical properties on adsorption behavior. The Langmuir model provided a better fit compared to the Freundlich model as indicated by the higher *R* ^2^ values (coefficient of determination) obtained for the linear regression analysis (0.98 and 0.97 for the Langmuir model vs 0.92 and 0.69 for the Freundlich model) (Table 2), suggesting an adsorption mechanism involving monolayer interactions on homogeneous active sites, in agreement with the synthesized TE-PLOT configuration featuring an internal porous coating with a high density of active adsorption sites. For 5-HT, a reasonable fit was also observed with the Freundlich model (*R* ^2^ = 0.92), suggesting the presence of additional heterogeneous interaction domains. This may be attributed to the aromatic structure of 5-HT, which can promote secondary interactions beyond those governing purely polar compounds such as GSH. This result may be influenced by the molecular structure of 5-HT, whose aromatic indole ring may promote secondary, nondominant hydrophobic interactions with the organic backbone of the thiol–ene polymer, resulting in a more heterogeneous adsorption behavior than that observed for GSH. In contrast, the highly polar structure of GSH favors retention through hydrogen bonding, dipole–dipole, and electrostatic interactions. The Scatchard analysis showed acceptable linearity for 5-HT (*R* ^2^ = 0.94), supporting a system characterized by binding sites with similar, although not identical, affinities. Overall, these results indicate that adsorption is primarily driven by a monolayer mechanism, with secondary contributions from heterogeneous interactions depending on analyte structure.

**Table 2:** Parameters Calculated by Fitting the Experimental Data Using the Langmuir, Freundlich, and Scatchard Models

Langmuir isotherm	5-HT	GSH
*Q*_max_ (ng cm^–1^)	362.3	509.2
KL (mL cm^–1^)	0.07	0.15
*R*_2_	0.9856	0.9734
Freundlich isotherm		
log(KF)	2.77	2.16
*n*	0.81	1.01
*R*_2_	0.9284	0.696
Scatchard isotherm		
KD (μg mL^–1^)	27.86	2.38
*R*_2_	0.9446	0.5336
*Q*_max_ (ng cm^–1^)	506.96	393.04

The *Q*_max_ values obtained from the Langmuir and Scatchard models indicated a satisfactory total amount of analyte adsorbed on the developed TE-PLOT column (approximately 500 ng per centimeter). These high *Q*_max_ values can be attributed to the introduced functional groups, which are capable of selectively attracting the analytes.

### Validation Results

The analytical method for the determination of selected neurotransmitters in different biological samples (plasma, mouse brain, and cells) using the developed online in-tube SPME method with TE-PLOT column was validated according to the main FDA guidelines employing a set of pooled samples. Validation was carried out for each matrix, and matrix-matched calibration curves were constructed for every type of biological sample. For plasma, a linear dynamic range of 2.5–100 ng mL^–1^ was established. except for GLU and DA. GLU exhibited a high endogenous concentration, whereas DA showed a very low endogenous level; therefore, the respective ranges were 1–10 μg mL^–1^ and 0.15–100 ng mL^–1^. Recovery and matrix effects were evaluated at three spiking levels (C_1_: 2.5; C_2_: 10; C_3_: 60 ng mL^–1^) (Table S1). For cells, the linear dynamic range was 158–10000 pmol per 10^6^ cells with recovery and matrix effects assessed at three fortification levels (C_1_: 150; C_2_: 500; C_3_: 10000 pmol per 10^6^ cells) (Table S2). For the mouse brain. a linear dynamic range of 0.03–1.3 μg g^–1^ was obtained, except for GABA due to its very low endogenous content (0.01–1.3 μg g^–1^). Recovery and matrix effects were evaluated at three spiking levels (C_1_: 0.03; C_2_: 0.5; C_3_: 1.3 μg g^–1^) (Table S3). In all cases. recoveries were quantitative (86–100%). and matrix effects were negligible across all investigated matrices (90–104%). Intraday and interday precision were assessed through recovery experiments. Intraday precision was evaluated by six replicate analyses (n = 6) performed on the same day, whereas interday precision was assessed over five consecutive days using five independently prepared TE-PLOT columns (n = 5), each analyzed in triplicate. Results were expressed as relative standard deviation (RSD). All RSD values were below 15%, meeting the acceptance criteria established by FDA guidelines (Table S4). The interday precision study not only demonstrated satisfactory day-to-day reproducibility of the method but also confirmed good batch-to-batch reproducibility in the preparation of the TE-PLOT columns. The linear dynamic ranges were established by considering the endogenous levels of neurotransmitters in the different matrices to enable accurate quantification. The coefficient of determination (*R* ^2^) for linear regression was greater than 0.97 in all cases. The LOQs were set at the lowest point of each calibration curve. Since a blank matrix could not be obtained, LODs were estimated from the LOQ based on the signal-to-noise (S/N) ratio and further confirmed by serial dilutions (Tables S1–S3). A key observation is that, in plasma samples, direct injection without IT-SPME failed to provide reliable detection, whereas the inclusion of the microextraction step enabled quantification at low ng mL^–1^ levels (Figure 7). Without the online solid-phase microextraction step, endogenous neurotransmitters in peripheral plasma could not be reliably detected (Figure S5). Figure S10 shows a chromatogram of the plasma sample and the neurotransmitters endogenously present within it.

**Figure 7:**
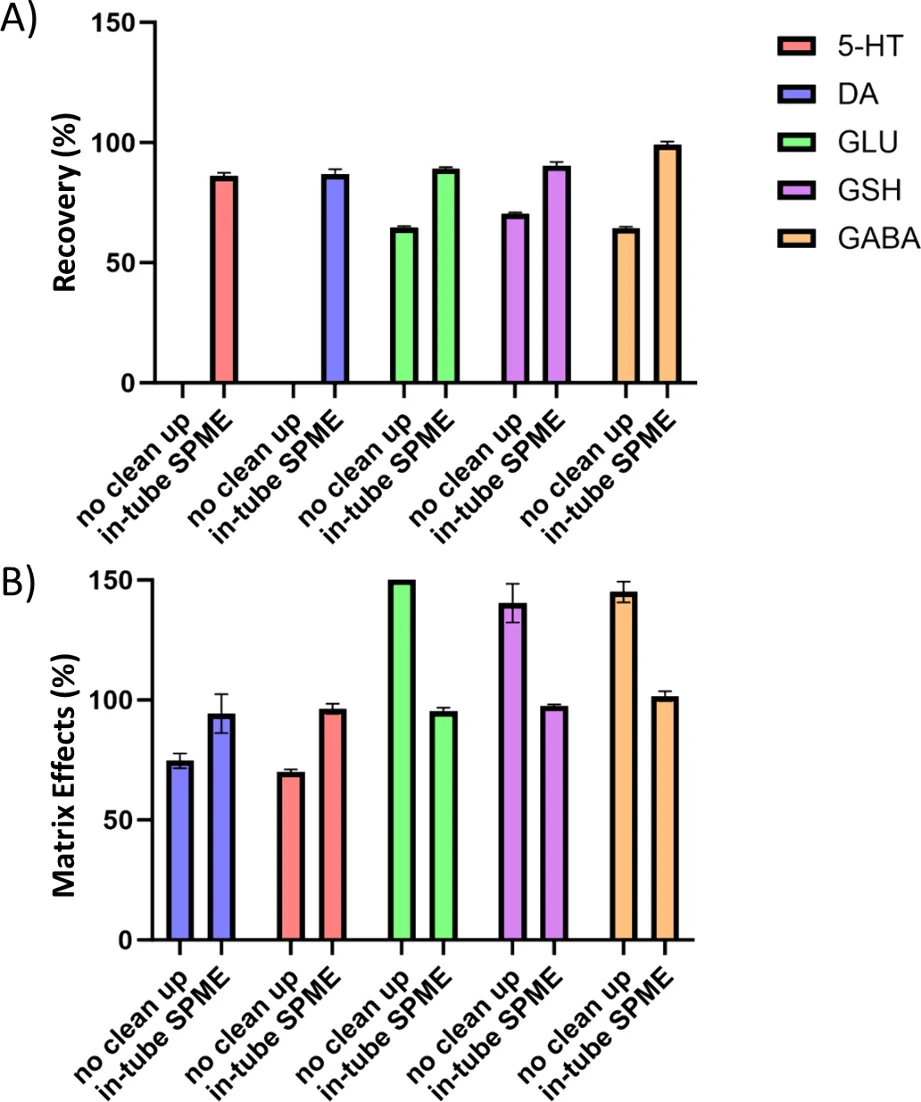
Histogram of (A) recovery and (B) matrix effect values for plasma samples with and without cleanup. The fortification level was 2.5 ng mL^–1^ for plasma.

In the other analyzed matrices (mouse brain and cells), this effect was not observed; however, without cleanup using the TE-PLOT, the matrix effect values were higher (Table S5).

### Analysis of the Real Samples

The developed method was successfully applied to plasma, human embryonic kidney (HEK-293) cells, and rat brain samples, demonstrating its applicability to biological matrices with markedly different biochemical compositions. The results of quantitative analysis are reported in Table 3.

**Table 3:** Results of the Quantitative Analysis of Different Neurotransmitters in the Various Analyzed Matrices

	Rat Brain μg g^–1^ ± dev. std			
5-HT	0.23 ± 0.01	0.121 ± 0.007	0.125 ± 0.007	0.144 ± 0.009
DA	0.24 ± 0.01	0.33 ± 0.02	0.36 ± 0.02	0.39 ± 0.02
GLU	0.141 ± 0.009	0.151 ± 0.008	0.20 ± 0.01	0.092 ± 0.006
GSH	2.2 ± 0.2	2.1 ± 0.1	2.1 ± 0.1	1.8 ± 0.1
GABA	0.0101 ± 0.0006	0.021 ± 0.001	0.031 ± 0.002	0.021 ± 0.001
	Cells nmol per 10^6^ cells ± dev. std			
GLU	7.9 ± 0.5	8.1 ± 0.5	7.8 ± 0.5	7.8 ± 0.5
GSH	6.0 ± 0.4	4.0 ± 0.2	3.2 ± 0.2	4.3 ± 0.3
	Plasma ng mL^–1^ ± dev. std			
5-HT	53 ± 3	93 ± 5	59 ± 3	83 ± 4
DA	4.3 ± 0.3	0.22 ± 0.02	3.5 ± 0.2	0.22 ± 0.01
GLU	7227 ± 361	6954 ± 348	6594 ± 330	6227 ± 311
GSH	61 ± 4	64 ± 4	61 ± 4	61 ± 4
GABA	8.7 ± 0.4	13.0 ± 0.6	8.2 ± 0.4	10.9 ± 0.5

For HEK-293 cells, only glutathione (GSH) and glutamate (GLU) were detected. This observation is consistent with the biological characteristics of these transformed human embryonic kidney cells, which are not neuronal cells and therefore do not physiologically synthesize monoaminergic neurotransmitters, such as dopamine, serotonin, or norepinephrine.^34,35^ Validation and quantification in this matrix were nevertheless performed, highlighting the versatility of the method, which can also be applied to complex biological matrices with low levels of specific neurotransmitters.

In rat brain samples, the measured concentrations of neurotransmitters were generally consistent with literature-reported ranges, supporting the reliability of the proposed method for quantitative analysis in complex nervous tissues and its applicability to neurochemical studies.^6,36^ Similarly, the concentrations determined in plasma, including serotonin, dopamine, glutamate, GABA, and glutathione, were within the ranges previously reported in metabolomics-based clinical studies.^37^ However, discrepancies in absolute values observed across studies may be attributed not only to biological variability but also to methodological differences, particularly in sample preparation and analyte recovery. The improved analytical performance of the present method likely reduces the underestimation effects associated with incomplete extraction or ion suppression. Furthermore, while several studies have investigated neurotransmitter alterations in neurological and psychiatric conditions,^38,39^ direct comparison of concentration values is often limited by differences in biological matrices (e.g., brain tissue vs plasma) and analytical platforms. In this context, the present approach provides a robust and minimally invasive tool for plasma-based neurochemical profiling with potential applications in clinical and translational research. Overall, the combination of a low sample volume requirement, high recovery, and negligible matrix effect positions this method as a highly efficient and reliable alternative to existing analytical workflows, offering improved accuracy and suitability for routine and large-scale studies.

### Comparative Analysis with Other Methods

The developed and validated method was compared with previously reported analytical approaches in terms of analytes and sample types, sample volume, enrichment strategy, sample preparation, analytical runtime, and overall performance (Table 4). The comparison highlights significant advantages regarding operational efficiency, reduced sample consumption, analytical sensitivity, and workflow automation. A major strength of the proposed approach is the combination of online in-tube SPME with LC-MS/MS, which requires only 50 μL of plasma while minimizing sample handling through automated online enrichment. This represents a clear improvement compared to several existing methods, which typically require larger plasma volumes and involve offline extraction procedures, often leading to increased variability and analyte loss.^40–42^ The use of online in-tube SPME overcomes the intrinsic limitations of liquid–liquid extraction (LLE)-based approaches or offline protein precipitation methods, which, although providing good recoveries, involve substantially longer analytical runtimes, typically ranging from 30 to 40 min.^3–6,43^ Compared with protocols involving laborious offline chemical derivatization (e.g., benzyl chloroformate, dansyl chloride, or FMOC), which introduce a high risk of experimental error and drastically extend total analysis time to more than 60–795 min,^8–12^ the proposed procedure does not require any derivatization step, reducing the total run time to only 20 min. Although methods based on simple offline dilution offer extremely short analysis times (6–10 min), they apply almost exclusively to purified biological fluids such as cerebrospinal fluid or dialysates, requiring minimal sample volumes but not providing any enrichment of the target analytes.^2,7^ In contrast, the present method requires only a small sample volume (50 μL), fully consistent with modern microextraction standards and comparable to other advanced analytical applications based on in-tube SPME.

**Table 4:** Comparison of the Developed and Validated Method with Previously Reported Analytical Methods Based on Analytes and Sample Types, Sample Volume, Enrichment Strategy, Sample Preparation, Analytical Runtime, And Overall Performance

compounds	sample	sample volume (mL or g)	sample prep.	online/offline	derivatization	runtime (min)	RE% (EM%)	LOD-LOQ (ng mL^–1^)	ref
DA, GABA, 5-HT, Gln, Glu	rat brain (hippocampus, amygdala)	180–250 g	liquid–liquid extraction; protein precipitation	offline	no	30	92.4–96.5 (n.r.)	0.001–1471.30 0.003–4413.90	^3^
GABA, DA, 5-HT, noradrenaline, 3,4-dihydroxyphenylacetic acid (DOPAC), homovanillic acid (HVA), 5-hydroxyindole-3-acetic acid (5-HIAA), epinephrine (E), tyramine (Tyn)	brain tissue	120–150 g	liquid–liquid extraction; protein precipitation	offline	no	30–35	94.04–107.53 (n.r.)	0.01–30 0.03–100	^4^
5-HT, DA	cerebrospinal fluid samples	10 mL	column-switching sistem	online	no	20	65–70 (86–120)	0.007–4.55 0.08–18.22	^44^
Trp, Tyr, 5-HT, 5-hydroxyindolacetic acid (5-HIAA), DA, acetylcholine (ACh), norepinephrine (NE), Glu, GABA	brain tissue	200–220 g	Liquid–Liquid extraction; protein precipitation	offline	no	40	85–115 (90–110)	n.r. 2.0–1000	^5^
BH4, DA, 5-HT, norepinephrine, epinephrine, Glu, GABA	mouse brain	0.01–0.04 g	liquid–liquid extraction; protein precipitation	offline	no	35	86.1–107.3 (86.5–108.1)	0.1–5 0.5–10	^6^
GABA, Glu, Gln, Trp, 5-HT, 5-HIAA, Phe, l-Tyr, DA, Levodopa (L-DOPA)	mouse hippocampus	0.01–0.02 g	liquid–liquid extraction; protein precipitation	offline	no	40	88–113 (85.1–114.9)	0.08–20 0.3–100	^43^
aspartic acid, Ser, Gly, Glu, GABA, norepinephrine, epinephrine, acetylcholine, DA, 5-HT, histamine, 1-metylhistamine	mouse cerebrospinal	0.002 mL	only dilution	offline	no	10	n.r.	n.r. 10–133	^2^
5-HT, 5-HIAA, melatonin, DA, l-DOPA, 3-methoxytyramine (3-MT), norepinephrine, epinephrine, acetylcholine, choline, GABA	cerebrospinal fluid microdialysis (MD)	0.015	only dilution	offline	no	6	86–115 (86.3–11.2)	0.003–0.31 0.01–1.03	^7^
Trp, L-5-HT, 5-HIAA, DA, DOPAC, 3-MT, GABA, Glu, homovanillic acid, l-kynurenine, kynurenic acid, 3-hydroxykynurenine, quinolinic acid	brain tissues- plasma	0.1 g 0.1 mL	Protein precipitation, Liquid–Liquid extraction, derivatization with 2-aminoethyl-trimethylammonium chloride	offline	yes	795	82 (n.r.)	1.8–2.2 2.5–2.8	^8^
Gln	brain tissues	0.002–0.005 g	derivatization with FMOC	offline	yes	61	n.r. (n.r.)	n.r. n.r.	^9^
DA, DOPAC, Trp, 5-HT, 5-HIAA, homovanillic acid, norepinephrine, l-kynurenine, kynurenic acid, 3-hydroxykynurenine, 3-hydroxyanthranilic acid	brain tissues, plasma	0.02–0.5 g 0.05 mL	derivatization with benzoyl chloride (BzCl)	offline	yes	40	80.3–102.6 (84.6–112.4)	0.006–3.0 0.02–10	^10^
arginine, tyrosine, tryptophan, phenylalanine, lysine, 3,4-dihydroxyphenylalanine, adenosine, cytidine, inosine, guanosine, uridine, pantothenic acid, dimethylsulfoniopropionate	surface coral reef seawater	100 mL	solid phase extraction (SPE) derivatization with benzoyl chloride (BzCl)	offline	yes	210	55–105 (80–120)	0.001–0.15 0.003–0.5	^11^
alanine, valine, leucine, isoleucine, tyrosine, tryptophan, phenylalanine, histidine, beta-alanine, taurine, DA, 5-HT, norepinephrine, putrescine, spermidine, spermine	urine, plasma	0.002 mL	protein precipitation, derivatization with dansyl chloride (Dns-Cl)	offline	yes	80	90–107 (85–115)	0.02–0.5 0.06–1.50	^12^
β-estradiol	plasma	0.05 mL (1.1)	in-tube SPME	online	no	15	94 (112)	0.008 0.18	^22^
5-HT, DA, Glu, GSH, GABA	plasma, rat brain, cells	0.05 mL (1.3)	in-tube SPME	online	no	20	86–100 (90–104)	0.05–0.71 0.15–1000	our work

From an analytical standpoint, the accuracy and sensitivity of the developed method are fully competitive: recoveries (RE% 86–100%) and matrix effects (ME% 90–104%) remain optimal and free from systematic bias when compared with column-switching systems, which typically exhibit lower extraction efficiencies (65–70%).^44^ In contrast, many previously published methodologies report moderate recoveries and significant matrix interferences, requiring complex correction strategies or internal standard normalization.^45,46^ Finally, the achieved limits of detection and quantification (LOD–LOQ: 0.05–0.71/0.15–1000 ng mL^–1^) ensure the robustness required for the simultaneous determination of neurotransmitters and related compounds in complex biological matrices (plasma, rat brain, and cell cultures), establishing the proposed approach as a fast, green, and high-throughput alternative for bioanalytical chemistry laboratories. A comparison was also performed with our previously developed method based on a monolithic column for the analysis of β-estradiol.^22^ Overall performance was comparable; however, the previously developed monolithic column did not allow sufficient retention of the analytes of interest in the present study, as it operated under different interaction mechanisms.

The TE-PLOT developed in this work was specifically designed as a multiplex platform for the simultaneous analysis of polar neurotransmitters, providing robust performance across analytes with markedly different physicochemical properties.

### Untargeted HRMS Analysis: Future Perspectives

To explore the broader applicability of the in-tube SPME approach beyond the targeted analytes, we performed an untargeted HRMS workflow. The TE-PLOT capillary was conditioned, loaded with peripheral plasma samples, and washed off-line. Subsequently, analyte elution was carried out online by coupling the capillary in series with the UHPLC-HRMS system, as described in the Supporting Information.

The untargeted analysis enabled the annotation of 60 compounds. According to an adapted confidence-level classification based on the Schymanski/MSI criteria,^47^ these annotations should be regarded as confidence level 2 identifications, as they were supported by accurate mass measurements and diagnostic MS/MS fragmentation patterns but were not confirmed using authentic analytical standards (Tables S6 and S7). Thirteen compounds were consistently detected in both positive- and negative-ionization modes. The annotated metabolites were distributed among four major chemical classes (Figure 8): acylcarnitines (Acetylcarnitine, Carnitine, Oleoylcarnitine); amino acids and peptides (cystine, arginine, citrulline, glutamine, histidine, lysine, ornithine, phenylalanine, tryptophan, tyrosine, asparagine, serine, threonine, leucine, valine, proline, phenylacetylglutamine, phenylalanylphenylalanine, and pyroglutamic acid); carbohydrates and organic acids (sucrose, threonic acid, and uric acid); lipids (glycerophosphocholine, glycerophosphoethanolamine, LPC 14:0, LPC 16:0, LPC 16:1, LPC 17:0, LPC 18:0, LPC 18:1, LPC 18:2, LPC 20:3, LPC 20:4, LPC 22:6, LPE 16:0, LPE 18:2, LPE 20:4, PC 16:0_18:1, PC 16:0_18:2, PC 16:0_20:4, PC 18:0_18:2, PC 34:1, PC 34:2, PC 36:2, PC 36:4, PC 38:4, PC 38:5, PC 38:6, PC 40:7, PC 40:8, PC 40:9, SM 34:1, and testosterone sulfate), together with a small group of miscellaneous metabolites, including 3-Indoleacrylic Acid, Creatine, Taurine, and Xylenesulfonate. The broad chemical diversity of the detected compounds highlights the versatility of the TE-PLOT extraction phase. Highly hydrophilic and zwitterionic analytes, including amino acids, carnitines, creatine, and sugars, are characterized by very low octanol/water partition coefficients (logP < 0) and preferentially partition into the water-enriched layer immobilized on the stationary phase surface. Consequently, their retention is favored by the HILIC-like extraction mechanism of the TE-PLOT phase. In addition, the stationary phase can promote hydrogen-bonding and electrostatic interactions. Amino acids and carnitines, such as acetylcarnitine, exist predominantly as zwitterions under the experimental conditions and can therefore establish strong electrostatic interactions while simultaneously acting as hydrogen-bond donors and acceptors. Although lipids would not traditionally be considered ideal candidates for HILIC-based retention because of their long hydrophobic acyl chains, their extraction can be rationalized by the dominant contribution of their polar head groups. Phosphatidylcholines (PCs and LPCs) and sphingomyelins contain a phosphocholine moiety comprising both negatively charged phosphate and positively charged quaternary ammonium functionalities, whereas LPE species contain a phosphoethanolamine group. These highly polar and often zwitterionic head groups strongly interact with the aqueous layer and undergo dipole–dipole and electrostatic interactions with the stationary phase, enabling efficient retention despite the presence of extended hydrophobic regions. Several detected metabolites, including threonic acid, uric acid, testosterone sulfate, and xylenesulfonate, contain strongly acidic functionalities such as carboxylate, sulfate, or sulfonate groups that can contribute to retention through electrostatic interactions. Furthermore, compounds such as threonic acid possess multiple hydroxyl groups capable of forming extensive hydrogen-bonding networks with the stationary phase. Aromatic metabolites, including 3-Indoleacrylic Acid and aromatic amino acids, may additionally benefit from dipole–dipole and induced-dipole interactions involving their conjugated π-electron systems, further stabilizing retention under the high-organic conditions employed for extraction. Overall, these findings suggest that the TE-PLOT in-tube SPME platform is not limited to the extraction of a single metabolite class but is capable of retaining structurally diverse compounds spanning a wide range of physicochemical properties. This broad analyte coverage highlights the potential of the proposed approach for future untargeted metabolomics and exposomics applications.

**Figure 8:**
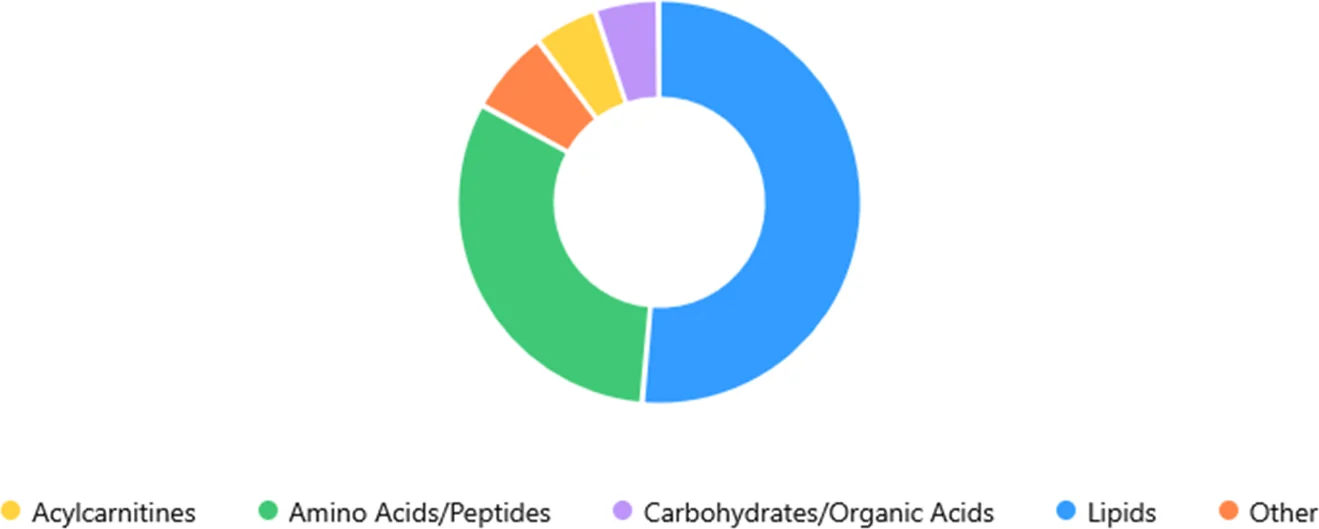
Distribution of metabolite classes identified by untargeted UHPLC-HRMS after extraction using the TE-PLOT in-tube SPME platform. Lipids (52%) and amino acids/peptides (32%) constituted the predominant classes, followed by acylcarnitines (5%), carbohydrates and organic acids (5%), and other metabolites (5%). Annotations were assigned at confidence level 2 according to the adapted Schymanski/MSI criteria.

## Conclusions

A thiol–ene porous layer open tubular column was successfully implemented as an online sample preparation tool for LC–MS/MS analysis of polar neurotransmitters. The developed platform enables direct, derivatization-free analysis by integrating cleanup and enrichment into a single step, reducing procedural complexity compared to conventional workflows. The method demonstrated robust analytical performance across multiple biological matrices, with recoveries ranging from 86% to 100% and matrix effects maintained within 90–104%, confirming effective mitigation of matrix interferences. The incorporation of the in-tube SPME step proved essential for enabling reliable detection of low-abundance neurotransmitters in plasma, where direct injection was not feasible. The physicochemical properties of the porous layer coating, including high permeability and multifunctional surface chemistry, support efficient interaction with low-molecular-weight polar analytes while maintaining operational stability over repeated analytical cycles. These results demonstrate the potential of the thiol–ene PLOT column for streamlined bioanalytical applications. The proposed strategy offers a practical alternative to conventional sample preparation strategies for neurotransmitter analysis and may be extended to other classes of small, polar metabolites requiring minimal sample handling.

## Supplementary Material



## Notes

Details on adsorption experiments; Material characterization by EDX and IR spectroscopy; Study of the reuse of the TE-PLOT; Thermodynamic studies of adsorption 5-HT and GSH onto TE-PLOT; The application of Langmuir, Freundlich, and Scatchard models are reported; Tables summarizing the analytical method validation; Comparison with a nonfunctionalized capillary; Real sample chromatogram (PDF)

## References

[1] Hook V., Kind T., Podvin S., Palazoglu M., Tran C., Toneff T., Samra S., Lietz C., Fiehn O. (2019). Metabolomics Analyses of 14 Classical Neurotransmitters by GC-TOF
with LC-MS Illustrates Secretion of 9 Cell-Cell Signaling Molecules
from Sympathoadrenal Chromaffin Cells in the Presence of Lithium. ACS Chem. Neurosci..

[2] Blanco M. E., Mayo O. B., Bandiera T., De Pietri Tonelli D., Armirotti A. (2020). LC-MS/MS Analysis of Twelve Neurotransmitters
and Amino
Acids in Mouse Cerebrospinal Fluid. J. Neurosci.
Methods.

[3] Alhusban A. A., Hammad A. M., Alzaghari L. F., Shallan A. I., Shnewer K. (2023). Rapid and
Sensitive HPLC-MS/MS Method for the Quantification of Dopamine, GABA,
Serotonin, Glutamine and Glutamate in Rat Brain Regions after Exposure
to Tobacco Cigarettes. Biomed. Chromatogr..

[4] Wang Y., Zheng R., Wu P., Wu Y., Huang L., Huang L. (2023). Determination of Multiple Neurotransmitters through LC-MS/MS to Confirm
the Therapeutic Effects of Althaea Rosea Flower on TTX-Intoxicated
Rats. Molecules.

[5] Xu H., Wang Z., Zhu L., Sui Z., Bi W., Liu R., Bi K., Li Q. (2018). Targeted Neurotransmitters
Profiling
Identifies Metabolic Signatures in Rat Brain by LC-MS/MS: Application
in Insomnia, Depression and Alzheimer’s Disease. Molecules.

[6] Kim T.-H., Choi J., Kim H.-G., Kim H. R. (2014). Quantification
of
Neurotransmitters in Mouse Brain Tissue by Using Liquid Chromatography
Coupled Electrospray Tandem Mass Spectrometry. J. Anal. Methods Chem..

[7] Helmschrodt C., Becker S., Perl S., Schulz A., Richter A. (2020). Development
of a Fast Liquid Chromatography-Tandem Mass Spectrometry Method for
Simultaneous Quantification of Neurotransmitters in Murine Microdialysate. Anal. Bioanal. Chem..

[8] Vondroušová J., Mikoška M., Syslová K., Böhmová A., Tejkalová H., Vacek L., Kodym P., Krsek D., Horáček J. (2019). Monitoring
of Kynurenine Pathway
Metabolites, Neurotransmitters and Their Metabolites in Blood Plasma
and Brain Tissue of Individuals with Latent Toxoplasmosis. J. Pharm. Biomed. Anal..

[9] Bheemanapally K., Ibrahim M. M. H., Briski K. P. (2020). Optimization of
Ultra-High-Performance
Liquid Chromatography-Electrospray Ionization-Mass Spectrometry Detection
of Glutamine-FMOC Ad-Hoc Derivative by Central Composite Design. Sci. Rep..

[10] Zheng X., Kang A., Dai C., Liang Y., Xie T., Xie L., Peng Y., Wang G., Hao H. (2012). Quantitative
Analysis
of Neurochemical Panel in Rat Brain and Plasma by Liquid Chromatography-Tandem
Mass Spectrometry. Anal. Chem..

[11] Garcia B.
M., Becker C. C., Weber L., Swarr G. J., Kido
Soule M. C., Apprill A., Kujawinski E. B. (2024). Benzoyl
Chloride Derivatization Advances the Quantification of Dissolved Polar
Metabolites on Coral Reefs. J. Proteome Res..

[12] Hsieh Y.-J., Chien K.-Y., Chang C.-M., Hung C.-Y., Li L., Chiang W.-F., Lee C.-C., Chang C.-H., Chang Y.-H., Yu J.-S. (2022). Development of a Method for Dansylation of Metabolites
Using Organic Solvent-Compatible Buffer Systems for Amine/Phenol Submetabolome
Analysis. Anal. Chim. Acta.

[13] Eeltink S., Svec F., Fréchet J. M.
J. (2006). Open-tubular Capillary
Columns with a Porous Layer of Monolithic Polymer for Highly Efficient
and Fast Separations in Electrochromatography. Electrophoresis.

[14] Zeeuw J. de, Luong J. (2002). Developments in Stationary
Phase Technology for Gas Chromatography. TrAC
Trends Anal. Chem..

[15] Webber K. G. I., Huang S., Truong T., Heninger J. L., Gregus M., Ivanov A. R., Kelly R. T. (2024). Open-Tubular
Trap Columns: Towards
Simple and Robust Liquid Chromatography Separations for Single-Cell
Proteomics. Mol. Omi..

[16] Hara T., Futagami S., Eeltink S., De Malsche W., Baron G. V., Desmet G. (2016). Very High Efficiency
Porous Silica
Layer Open-Tubular Capillary Columns Produced via in-Column Sol-Gel
Processing. Anal. Chem..

[17] Nechvátalová M., Urban J. (2022). Current Trends
in the Development of Polymer-based Monolithic Stationary
Phases. Anal. Sci. Adv..

[18] Liu Z., Ou J., Zou H. (2016). Click Polymerization
for Preparation of Monolithic
Columns for Liquid Chromatography. TrAC Trends
Anal. Chem..

[19] Lyu H., Sun H., Zhu Y., Wang J., Xie Z., Li J. (2020). A Double-Recognized
Aptamer-Molecularly Imprinted Monolithic Column for High-Specificity
Recognition of Ochratoxin A. Anal. Chim. Acta.

[20] Zhang Q., Zhang X., Yang B., Liu S., Wen M., Bao L., Jiang L. (2022). Development of a Highly
Efficient In-tube Solid-phase
Microextraction System Coupled with UHPLC-MS/MS for Analyzing Trace
Hydroxyl Polycyclic Aromatic Hydrocarbons in Biological Samples. J. Sep. Sci..

[21] Yang Y., Huang Y., Wu Z., Shi R., Chen Z., Ruan G. (2022). Porous Capillary Monolithic Column Coupled with Ultrahigh Performance
Liquid Chromatography-Tandem Mass Spectrometry for Fast and Effective
Separation and Determination of Estrogens. Anal.
Chim. Acta.

[22] Cerrato A., Aita S. E., Cavaliere C., Laganà A., Montone C. M., Piovesana S., Taglioni E., Capriotti A. L. (2024). Preparation
of Monolith for Online Extraction and LC-MS Analysis of β-Estradiol
in Serum Via a Simple Multicomponent Reaction. Anal. Chem..

[23] Piovesana S., Aita S. E., Cavaliere C., Cerrato A., Laganà A., Montone C. M., Taglioni E., Capriotti A. L. (2024). Validation
of a Global Method for the Simultaneous Analysis of Polar and Non-Polar
Pesticides by Online Extraction and LC-MS/MS. Anal. Chim. Acta.

[24] Guiochon G. (2007). Monolithic
Columns in High-Performance Liquid Chromatography. J. Chromatogr. A.

[25] Ahmed M. A., Felisilda B. M. B., Quirino J. P. (2019). Recent Advancements in Open-Tubular
Liquid Chromatography and Capillary Electrochromatography during 2014–2018. Anal. Chim. Acta.

[26] Kaufmann A. (2009). Validation
of Multiresidue Methods for Veterinary Drug Residues; Related Problems
and Possible Solutions. Anal. Chim. Acta.

[27] Durham O. Z., Norton H. R., Shipp D. A. (2015). Functional Polymer
Particles via
Thiol-Ene and Thiol-Yne Suspension “Click” Polymerization. RSC Adv..

[28] Wang Z., Xiao C., Wu C., Han H. (2000). High-Performance Polyethylene
Glycol-Coated Solid-Phase Microextraction Fibers Using Sol-Gel Technology. J. Chromatogr. A.

[29] Naga N., Sato M., Mori K., Nageh H., Nakano T. (2020). Synthesis
of Network Polymers by Means of Addition Reactions of Multifunctional-Amine
and Poly­(Ethylene Glycol) Diglycidyl Ether or Diacrylate Compounds. Polymers (Basel)..

[30] Schick B. W., Uhl M., Al-Shakran M., Bansmann J., Riedel S., Zhao-Karger Z., Jacob T. (2025). Diethylene Glycol Diethyl Ether as Electrolyte Solvent for Reversible
Electrochemical Magnesium Plating. ChemElectroChem..

[31] Svec F. (2004). Preparation
and HPLC Applications of Rigid Macroporous Organic Polymer Monoliths. J. Sep. Sci..

[32] Qiao L., Lv W., Chang M., Shi X., Xu G. (2018). Surface-Bonded Amide-Functionalized
Imidazolium Ionic Liquid as Stationary Phase for Hydrophilic Interaction
Liquid Chromatography. J. Chromatogr. A.

[33] Buszewski B., Noga S. (2012). Hydrophilic Interaction
Liquid Chromatography (HILIC)—a Powerful
Separation Technique. Anal. Bioanal. Chem..

[34] Shaw K., Turner J., Del Mar C. (2002). Are Tryptophan and 5-Hydroxytryptophan
Effective Treatments for Depression? A Meta-Analysis. Aust. New Zeal. J. Psychiatry.

[35] Jiang J., Lv Z., Gu Y., Li J., Xu L., Xu W., Lu J., Xu J. (2010). Adult Rat
Mesenchymal Stem Cells Differentiate into
Neuronal-like Phenotype and Express a Variety of Neuro-Regulatory
Molecules in Vitro. Neurosci. Res..

[36] Brundu S., Nencioni L., Celestino I., Coluccio P., Palamara A. T., Magnani M., Fraternale A. (2016). Validation
of a Reversed-Phase High
Performance Liquid Chromatography Method for the Simultaneous Analysis
of Cysteine and Reduced Glutathione in Mouse Organs. Oxid. Med. Cell. Longev..

[37] Pan J.-X., Xia J.-J., Deng F.-L., Liang W.-W., Wu J., Yin B.-M., Dong M.-X., Chen J.-J., Ye F., Wang H.-Y. (2018). Diagnosis of Major Depressive Disorder Based on Changes
in Multiple Plasma Neurotransmitters: A Targeted Metabolomics Study. Transl. Psychiatry.

[38] Zhong Z., Zhou S., Xiang B., Wu Y., Xie J., Li P. (2021). Association of Peripheral Plasma Neurotransmitters
with Cognitive
Performance in Chronic High-Altitude Exposure. Neuroscience.

[39] Qian Q.-Q., Tan Q.-Q., Sun D., Lu Q., Xin Y.-Y., Wu Q., Zhou Y., Liu Y.-X., Tian P.-C., Liu Z.-S. (2022). A Pilot
Study on Plasma and Urine Neurotransmitter Levels in Children with
Tic Disorders. Brain Sci..

[40] Zhu M., Liu G., Chen H., Ma W., Cui L., Nimmu N. V., Hou H., Hu Q., Zhang Y. (2023). Analytical Strategies in Neurotransmitter
Measurements: A Mini Literature Review. Biomed.
Chromatogr..

[41] Wei M., Liu Y., Pi Z., Yue K., Li S., Hu M., Liu Z., Song F., Liu Z. (2019). Investigation of Plasma Metabolomics
and Neurotransmitter Dysfunction in the Process of Alzheimer’s
Disease Rat Induced by Amyloid Beta 25–35. RSC Adv..

[42] Lai Y., Liu C.-W., Chi L., Ru H., Lu K. (2021). High-Resolution
Metabolomics of 50 Neurotransmitters and Tryptophan Metabolites in
Feces, Serum, and Brain Tissues Using UHPLC-ESI-Q Exactive Mass Spectrometry. ACS Omega.

[43] Sun B., Xia Z., Wang C., Wu M., Tie R., Ru Y., Meng Y., Bai L., Zhang Y., Jia P. (2025). Development of a Non-Derivatized
LC-MS/MS Method for Simultaneous
Quantification of Ten Neurotransmitters in Five Neuroinflammatory
Mouse Models. Talanta Open.

[44] Suominen T., Uutela P., Ketola R. A., Bergquist J., Hillered L., Finel M., Zhang H., Laakso A., Kostiainen R. (2013). Determination of Serotonin and Dopamine Metabolites
in Human Brain Microdialysis and Cerebrospinal Fluid Samples by UPLC-MS/MS:
Discovery of Intact Glucuronide and Sulfate Conjugates. PLoS One.

[45] Veselova I. A., Sergeeva E. A., Makedonskaya M. I., Eremina O. E., Kalmykov S. N., Shekhovtsova T. N. (2016). Methods
for Determining Neurotransmitter Metabolism
Markers for Clinical Diagnostics. J. Anal. Chem..

[46] Reveil L., Lasaosa M., Furman A., Iqbal O’Meara A. M., Halquist M. S. (2026). Validation and Quantitation of Six Neurotransmitters
in Rat Plasma and Brain Using Liquid Chromatography Quadrupole-Time-of-Flight
Mass Spectrometry. J. Pharm. Biomed. Anal..

[47] Schymanski E. L., Jeon J., Gulde R., Fenner K., Ruff M., Singer H. P., Hollender J. (2014). Identifying
Small Molecules via High
Resolution Mass Spectrometry: Communicating Confidence. Environ. Sci. Technol..

