# Supplementary material for: Thiol–Ene Click-Derived Porous-Layer Open-Tubular Column for Online Purification and Multiplex LC–MS/MS Analysis of Neurotransmitters
Source: Anal Chem. 2026 Aug 24;98(35):26089–103. doi: 10.1021/acs.analchem.6c04601 (PMC13564031; doi:10.1021/acs.analchem.6c04601)
Supplement: Supplementary file 1 [file ac6c04601_si_001.pdf]

Supporting Information

Thiol–Ene Click-Derived Porous-Layer Open-Tubular Column for Online Purification and Multiplex LC–MS/MS Analysis of Neurotransmitters

Enrico Taglioni[1,2], Andrea Cerrato[1,2], Aldo Laganà[1,2], Carmela Maria Montone[1,2]*, Anna Laura Capriotti[1,2]

[1]Department of Chemistry, Sapienza University of Rome, Piazzale Aldo Moro 5, 00185 Rome, Italy

[2]Interuniversity Consortium INBB – Biostructures and Biosystems National Institute, Via dei Carpegna 19, 00165, Rome, Italy

*Corresponding author: Carmela Maria Montone

Department of Chemistry

Sapienza university of Rome

Piazzale Aldo Moro 5

00185 Rome, Italy

E-mail: carmelamaria.montone@uniroma1.it

tel: +39 06 4991 3945

Table of contents:

Experimental Section

Adsorption esperiments

UHPLC-HRMS exploratory untargeted metabolomics analysis

List of Figures:

Figure S1: (A-B) Representative examples of cross-sections obtained after mechanical cutting of the monolithic capillary column. The images show partial fracture, deformation, and detachment of the fused-silica capillary from the surrounding PEEK support, illustrating the difficulties associated with sample preparation for SEM characterization. (C) High-magnification SEM image of the coating selected for surface EDX analysis.

Figure S2: Microanalysis by EDS of the TE-PLOT column.The analysis shows the presence of carbon (53.28 wt%), oxygen (27.67 wt%), sulfur (12.20 wt%), and silicon (6.85 wt%). The sulfur signal confirms the successful thiol (–SH) functionalization of the porous layer structure. Chromium was set to zero in the quantitative analysis, as the sample had been previously chromium-coated prior to EDS measurement.

Figure S3: IR spectra of the monomers used for the synthesis of the TE-PLOT column: A) pentaerythritol tetrakis(3-mercaptopropionate), B) N,N′ methylenebis(acrylamide).

Figure S4: Backpressure as a function of different operating flow rates (0.03, 0.05, 0.1, 0.15, 0.2 mL min^{-1}). The linear relationship between flow rate and pressure indicates laminar flow conditions, allowing the pressure drop to be calculated from the slope of the linear fit.

Figura S5: A mixture of neurotransmitters (20 ng mL^{-1}) was injected into the in-tube SPME system using non-functionalized PEEK-sil tubing; from top to bottom, 5-HT (black), DA (red), GLU (green), GSH (blue), and GABA (yellow).

Figure S6: TE-PLOT column stability tests: (A) pH tolerance (pH 3-10), (B) repeated use (1–250 consecutive injections), (C) storage period (0–12 months)..

Figure S7: Thermodynamic adsorption of 5-HT (A) and GSH (B) in the TE-PLOT column.

Figure S8: Plots illustrating the application of the Langmuir (A, B) and Freundlich (C, D) models to the experimental rebinding data for 5-HT (A, C) and GSH (B, D), as described in the

Supplementary Information. The linear fitting equations and the corresponding coefficients of determination (R^2) are reported at the top of each panel.

Figure S9: Scatchard fitting of experimental data of the adsorption isotherms, for 5-HT (A), and GSH (B).

Figura S10: Chromatogram of the plasma sample injected into the in-tube SPME system, showing the endogenous levels of neurotransmitters: from top to bottom, 5-HT (black), DA (red), GLU (green), GSH (blue), and GABA (yellow).

List of Tables:

Table S1: Validation results for the plasma matrix at three different concentration levels (C1: 2.5; C2: 10; C3: 60 ng mL^{-1}), linear dynamic range, regression coefficients, LOD, and LOQ.

Table S2: Validation results for the cell matrix at three different concentration levels (C1: 150; C2: 500; C3: 10,000 pmol per 10^6 cells), linear dynamic range, regression coefficients, LOD, and LOQ.

Table S3: Validation results for the mouse brain matrix at three different concentration levels (C1: 0.03; C2: 0.5; C3: 1.3 µg g^{-1}), linear dynamic range, regression coefficients, LOD, and LOQ.

Table S4: Intraday and interday precision (RSD%) were evaluated by performing recovery experiments (n = 6) on the same day and over six consecutive days at C_2 (10 ng mL^{-1} for plasma; 500 pmol per 10^6 cells for cells; 0.5 µg g^{-1} for mouse brain).

Table S5: Recovery values and matrix effect for different biological matrices compared with and without clean-up. Spiking level for plasma: 2.5 ng mL^{-1}; for cells: 150 pmol per 10^6 cells; for mouse brain: 0.5 µg g^{-1}.

Table S6: Diagnostic MS/MS fragmentation data for the tentatively identified metabolites in positive mode.

Table S7: Diagnostic MS/MS fragmentation data for the tentatively identified metabolites in negative mode.

Adsorption experiments

To evaluate the adsorption performance and capacity of the monolithic@SH column, off-line loading experiments were carried out using 5-HT and GSH standard solutions under previously optimized conditions (water/acetonitrile 50/50, v/v, containing 0.1% NH_4OH). After loading, the eluates were collected and analyzed by UHPLC-MS/MS according to the established analytical procedure. Standard solutions at increasing concentrations (5–200 µg mL⁻¹) were introduced into the monolithic column at a flow rate of 0.1 mL min⁻¹.

The equilibrium adsorption capacity, Q_e (µg cm⁻¹), was determined according to Equation (1):

$$Qe = \frac{(C_0 - Ce)V}{L} \qquad \text{Eq. (1)}$$

where C_0 (µg mL⁻¹) is the initial concentration of 5-HT or GSH, C_e (µg mL⁻¹) is the equilibrium concentration, V (mL) represents the solution volume, and L (cm) corresponds to the length of the monolithic column. To elucidate the adsorption mechanism, the experimental data were fitted to both the Langmuir and Freundlich isotherm models. The Langmuir model is expressed by Equation (2):

$$Q_e = \frac{Q_{max} K_L C_e}{1 + K_L C_e} \qquad \text{Eq. (2)}$$

In this equation, Q_e denotes the amount of 5-HT or GSH adsorbed per centimeter of monolithic column, C_e is the equilibrium concentration in the supernatant, K_L is the Langmuir constant related to the binding affinity, and Qmax represents the maximum adsorption capacity. The parameters Qmax and K_L were determined from the linear regression plot of C_e/Q_e versus C_e.

The Freundlich isotherm was applied using Equation (3):

$$Q_e = K_F C_e^{\frac{1}{n}} \qquad \text{Eq. (3)}$$

where K_F is the Freundlich constant associated with adsorption capacity, and 1/n reflects the heterogeneity of the adsorption surface. The constants K_F and n were obtained from the linear regression of log Q_e versus log C_e. All adsorption experiments were performed in duplicate.

Additionally, Scatchard analysis was conducted to estimate the theoretical number of binding sites, using Equation (4):

$$\frac{Q_e}{C_e} = \frac{Q_{max} - Q_e}{K_D} \qquad \text{Eq. (4)}$$

where K_D is the Scatchard dissociation constant.

UHPLC-HRMS exploratory untargeted metabolomics analysis

Metabolite separation was carried out using a Vanquish binary pump H (Thermo Fisher Scientific, Bremen, Germany), equipped with a thermostated autosampler and column compartment, on Luna™ Polar Pesticide analytical column (2.1 × 100 mm, 3 µm particle size; Phenomenex) at 57 °C with a flow rate of 150 µL min–1, using water (A) and acetonitrile (B), each containing 0.1% (v/v) formic acid. The chromatographic conditions and gradient were as already outilined in the Materials and Methods section of the manuscript. The UHPLC system was coupled to a Q Exactive hybrid quadrupole-Orbitrap mass spectrometer (Thermo Fisher Scientific). Samples were analyzed in the positive ion mode (ESI+) and negative ion mode (ESI-) with the following HESI parameters: spray voltage at 2800 V, auxiliary gas heater temperature at 350 °C, sheath gas at 20 (arbitrary units), auxiliary gas at 10 (arbitrary units), sweep gas at 0 (arbitrary units), and S-Lens RF level was 55 (%). Top 5 data-dependent acquisition mode (DDA) was employed in the range 200-900 m/z at a resolution of 17,500 (FWHM, m/z 200), the AGC target at 100,000, maximum injection time at 50 ms, isolation window at 2.0 m/z, and higher-energy collisional dissociation (HCD) was performed at 30% normalized collision energy. Dynamic exclusion was set to 3 s. The mass spectrometer was calibrated before analysis using a calibration solution provided by the manufacturer (external calibration). The TE-PLOT column was originally designed as an online SPE clean-up procedure; however, evaluation of the applicability of the synthesized capillary stationary phase to the extraction of a broad range of polar analytes required its coupling with HRMS. Due to hardware limitations of the available UHPLC system (Vanquish, Thermo Fisher Scientific), which was not equipped with the valve configuration necessary to perform the conditioning, sample loading, washing, and elution steps in a fully automated online format, the capillary SPE column could not be operated under the intended setup. Consequently, plasma sample

pretreatment was carried out offline using Hamilton syringes to simulate the complete SPE workflow. After extraction, retained analytes were eluted and directed to an Orbitrap high-resolution mass spectrometer through a diverter valve connecting the chromatographic system to the MS detector. This alternative configuration enabled the characterization of the adsorption capability and selectivity of the synthesized stationary phase toward structurally diverse polar compounds, while overcoming the instrumental constraints that prevented implementation of the fully online methodology. The diverter valve started at position 2 (1-6), switched after 1 minute to position 1 (1-2) to enable analyte elution from the TE-PLOT column directly into the chromatographic column and then into MS. After 20 minutes the diverter valve switched back to position 2. All samples were collected in duplicate for each polarity. Raw data files were acquired with Xcalibur software (version 3.1, Thermo Fisher Scientific), and then preprocessed with Compound Discoverer (v. 3.1, Thermo, Waltham, USA) using a customized block-diagram data processing workflow specifically dedicated to polar metabolites. Briefly, the first processing node comprises the selection of spectra, then chromatographic alignment is carried out using an adaptive curve approach, allowing a maximum retention time shift of 0.2 min. The third node employs the "detect compound" tool, where precursor ions are extracted based on the selected possible adducts. Subsequently, the "group compound" function groups adducts and isotopic peaks using a mass tolerance of 5 ppm and a retention time tolerance of 0.2 min. The fill gaps tool represents a key stage, as it automatically corrects potential peak detection or alignment inaccuracies by searching for missing peaks and, eventually, background compounds are removed if the sample to blank area ratio is less than 5. Moreover, parameters for the predict composition tool were adapted to polar metabolite analysis. The extracted chromatographic masses were then filtered to eliminate background compounds detected in blank samples and features lacking fragmentation data. Unknown metabolites were then annotated based on retention time (RT), high-resolution accurate mass measurements, MS/MS fragmentation patterns, and spectral matching against open-source databases (e.g., mzCloud, MassBank) or previously reported literature data.

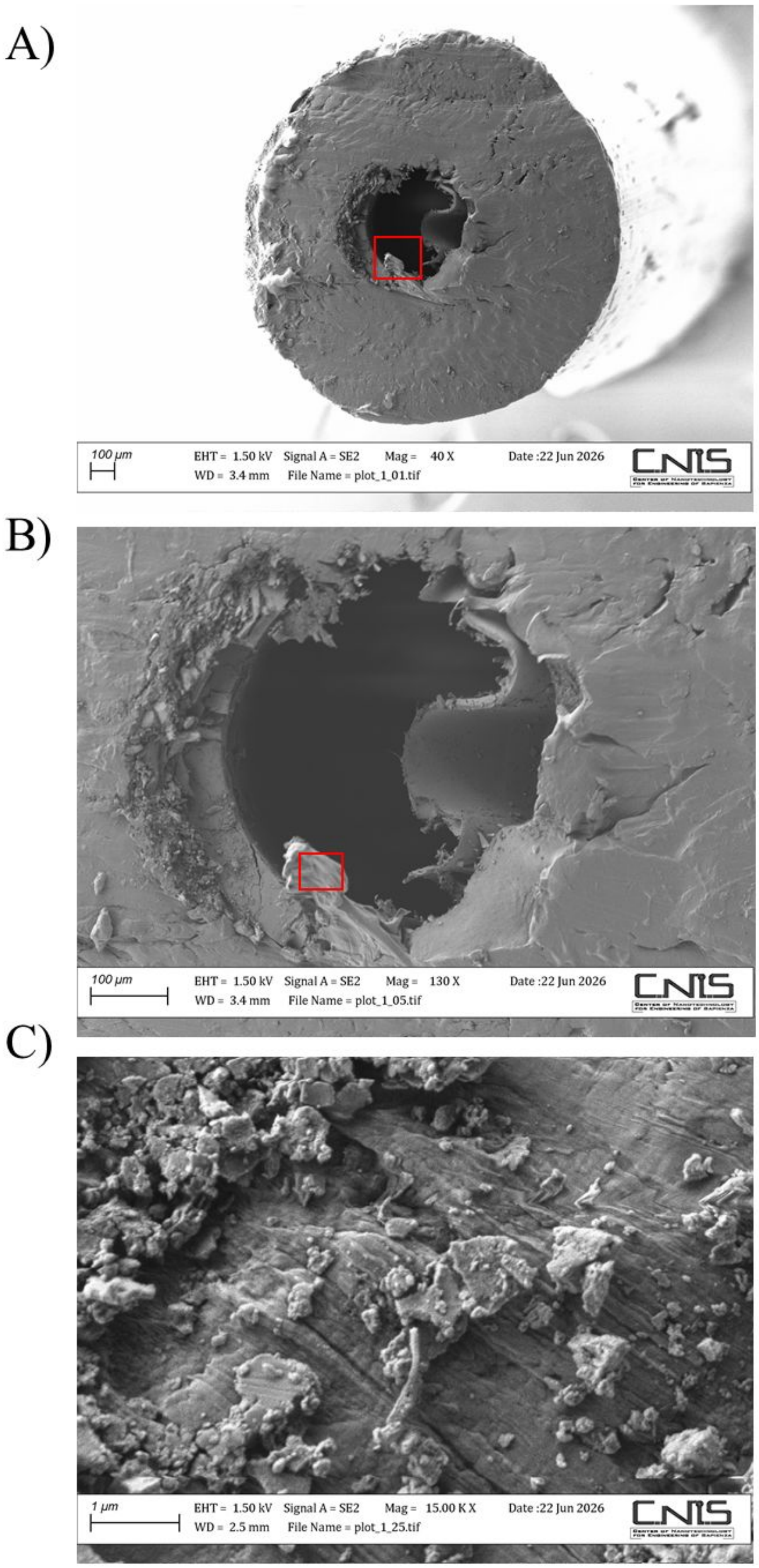

Figure S1: (A-B) Representative examples of cross-sections obtained after mechanical cutting of the monolithic capillary column. The images show partial fracture, deformation, and

detachment of the fused-silica capillary from the surrounding PEEK support, illustrating the difficulties associated with sample preparation for SEM characterization. (C) High-magnification SEM image of the coating selected for surface EDX analysis..

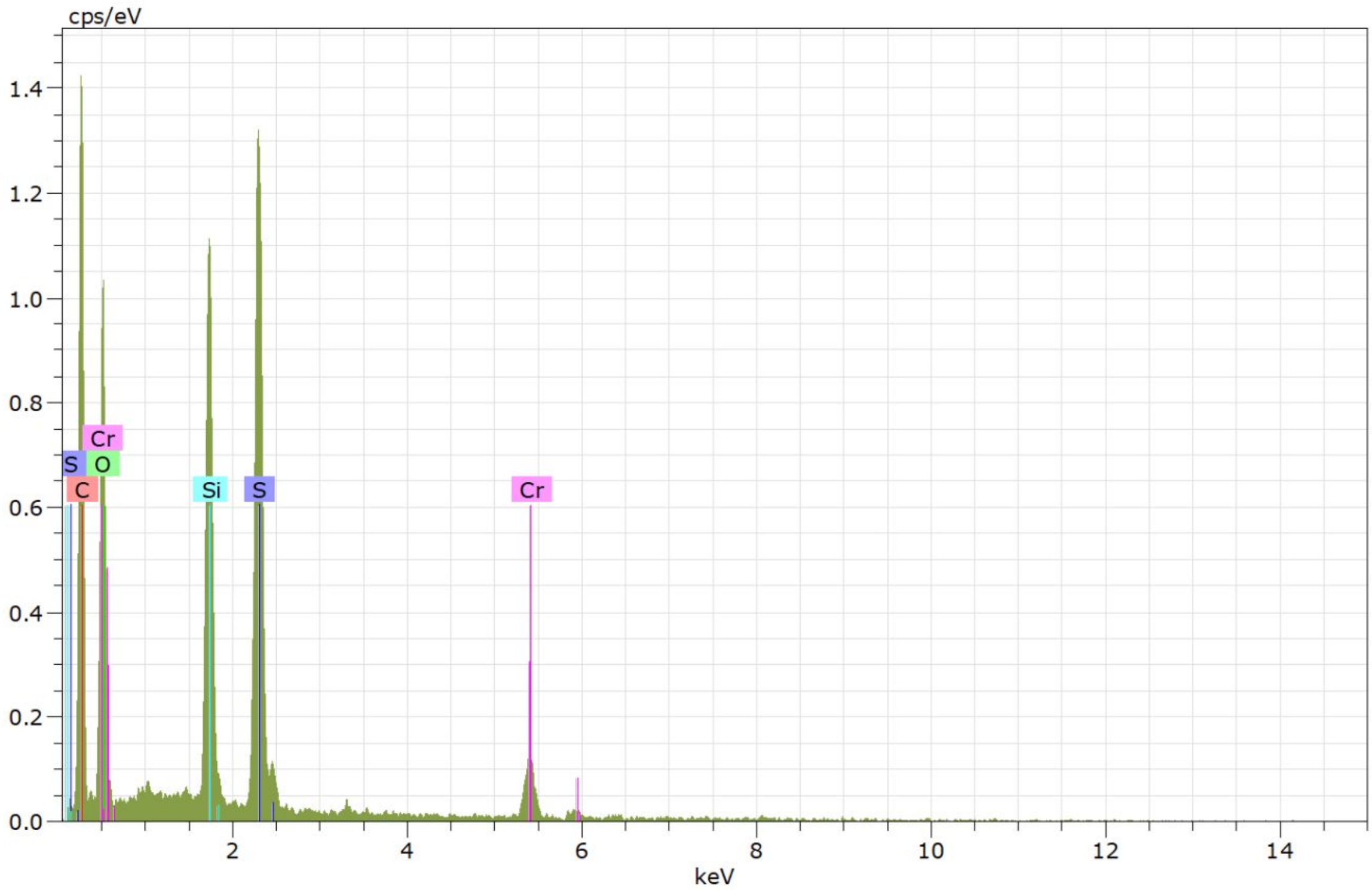

Figure S2: Microanalysis by EDS of the TE-PLOT column.The analysis shows the presence of carbon (53.28 wt%), oxygen (27.67 wt%), sulfur (12.20 wt%), and silicon (6.85 wt%). The sulfur signal confirms the successful thiol (–SH) functionalization of the porous layer structure. Chromium was set to zero in the quantitative analysis, as the sample had been previously chromium-coated prior to EDS measurement..

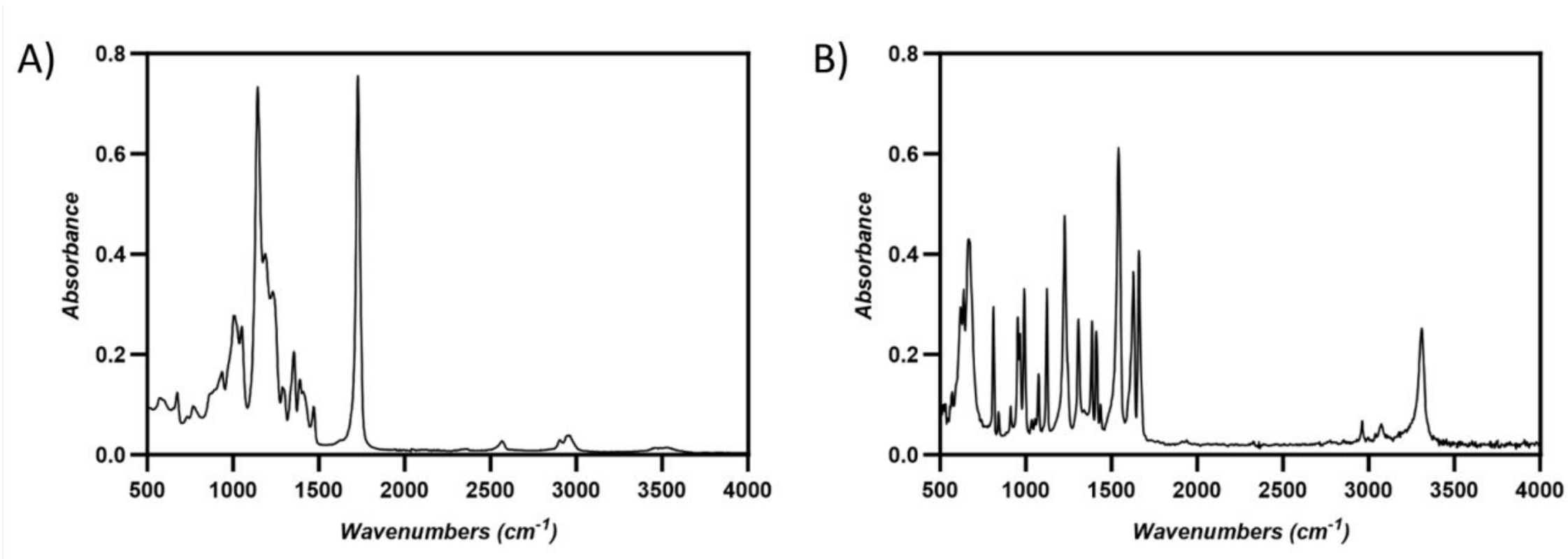

Figure S3: IR spectra of the monomers used for the synthesis of the TE-PLOT column: A) pentaerythritol tetrakis(3-mercaptopropionate), B) N,N′ methylenebis(acrylamide).

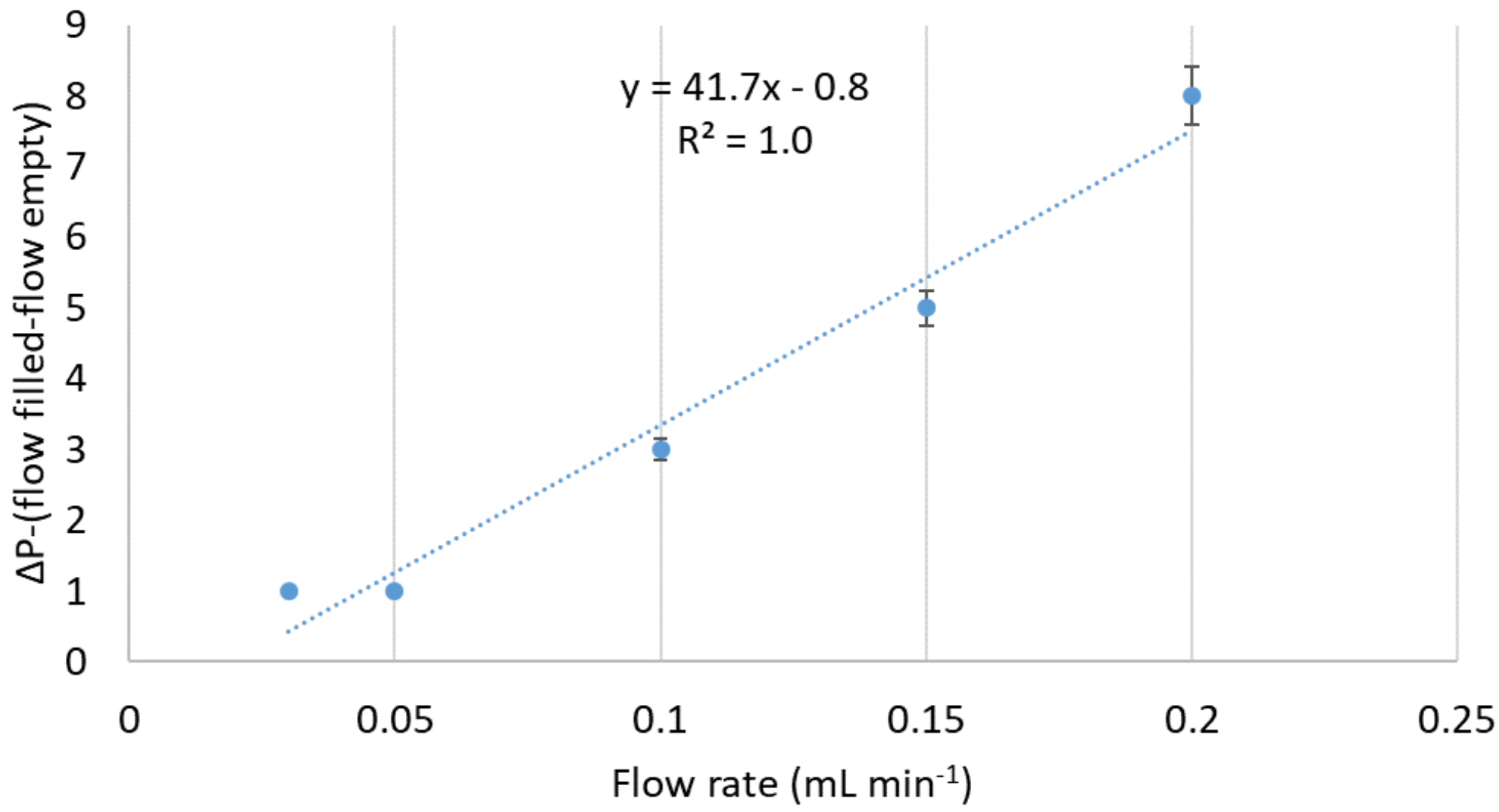

Figure S4: Backpressure as a function of different operating flow rates (0.03, 0.05, 0.1, 0.15, 0.2 mL min^{-1}). The linear relationship between flow rate and pressure indicates laminar flow conditions, allowing the pressure drop to be calculated from the slope of the linear fit.

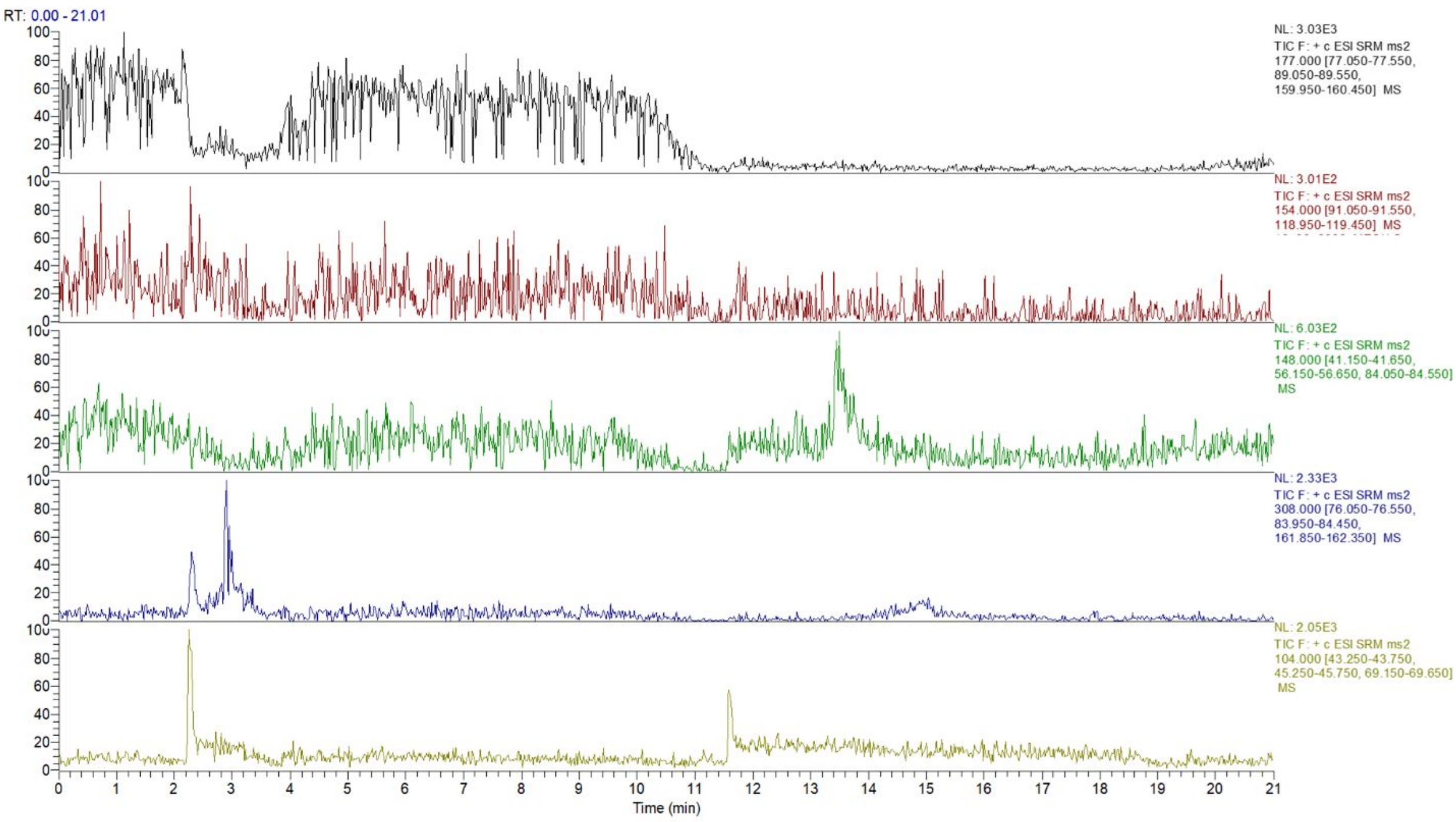

Figura S5: A mixture of neurotransmitters (20 ng mL^{-1}) was injected into the in-tube SPME system using non-functionalized PEEK-sil tubing; from top to bottom, 5-HT (black), DA (red), GLU (green), GSH (blue), and GABA (yellow).

A)

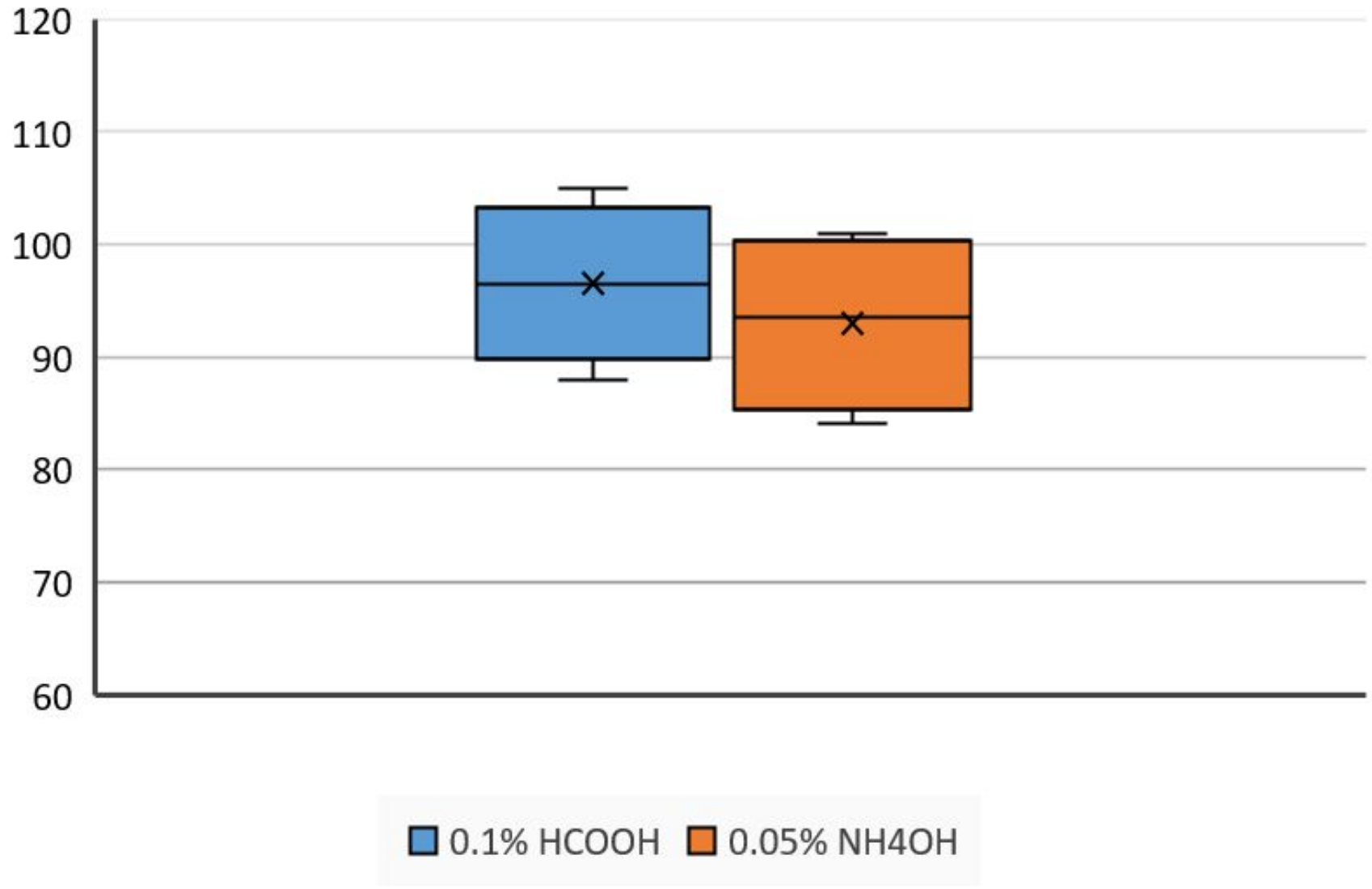

120
110
100
90
80
70
60
0.1% HCOOH
0.05% NH4OH

B)

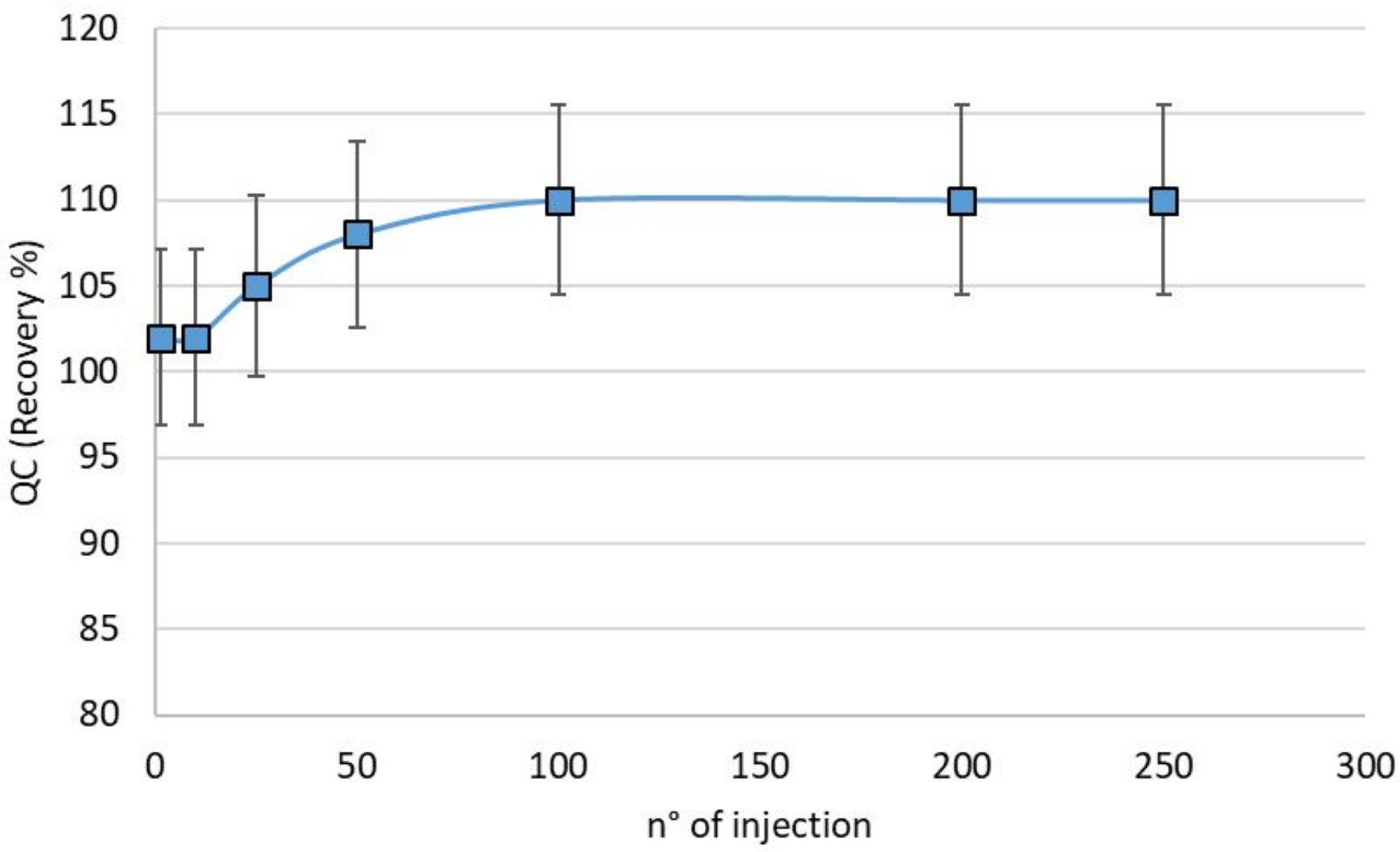

QC (Recovery %)
120
115
110
105
100
95
90
85
80
0
50
100
150
200
250
300
n° of injection

C)

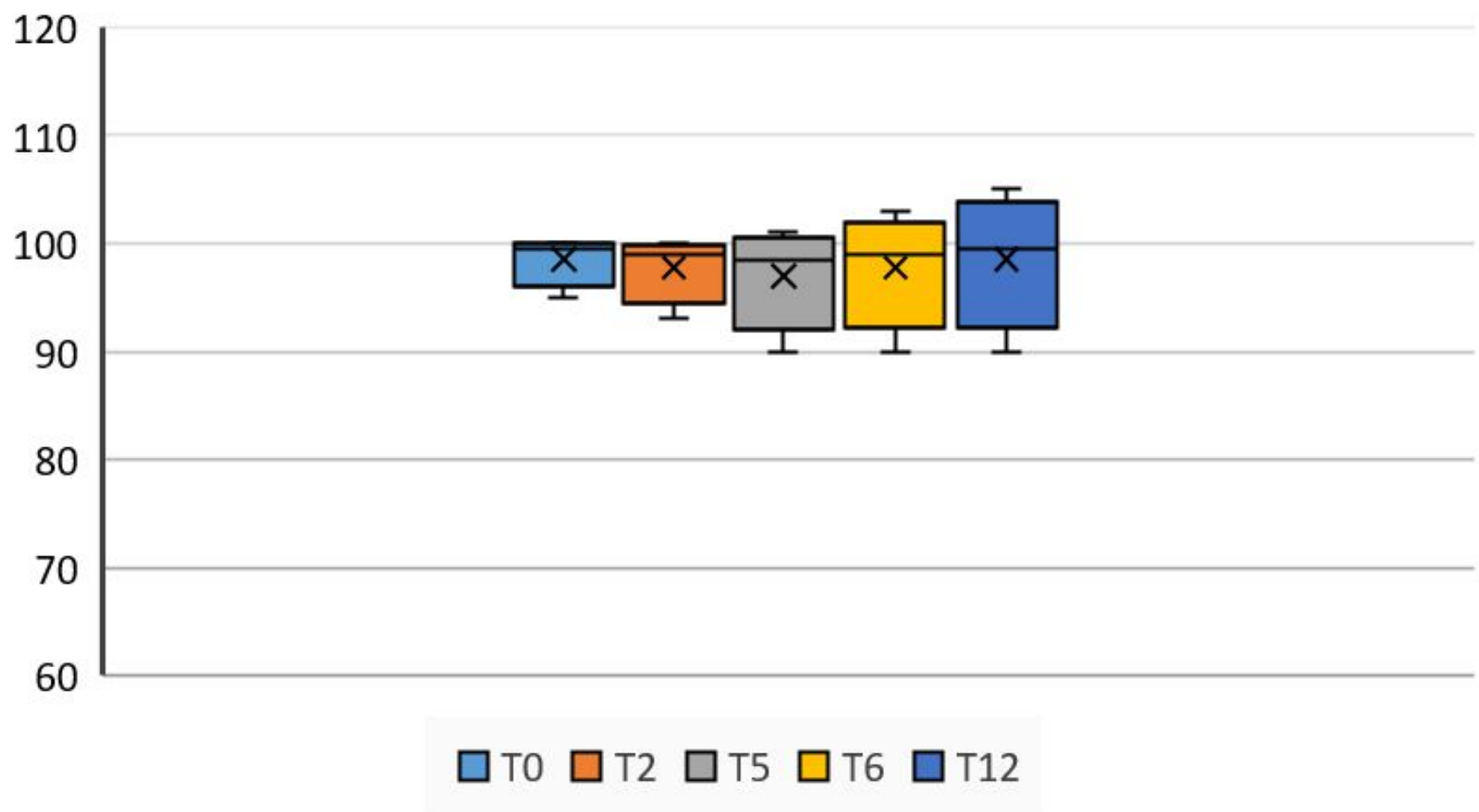

120
110
100
90
80
70
60
T0
T2
T5
T6
T12

Figure S6: TE-PLOT column stability tests: (A) pH tolerance (pH 3-10), (B) repeated use (1–250 consecutive injections), (C) storage period (0–12 months).

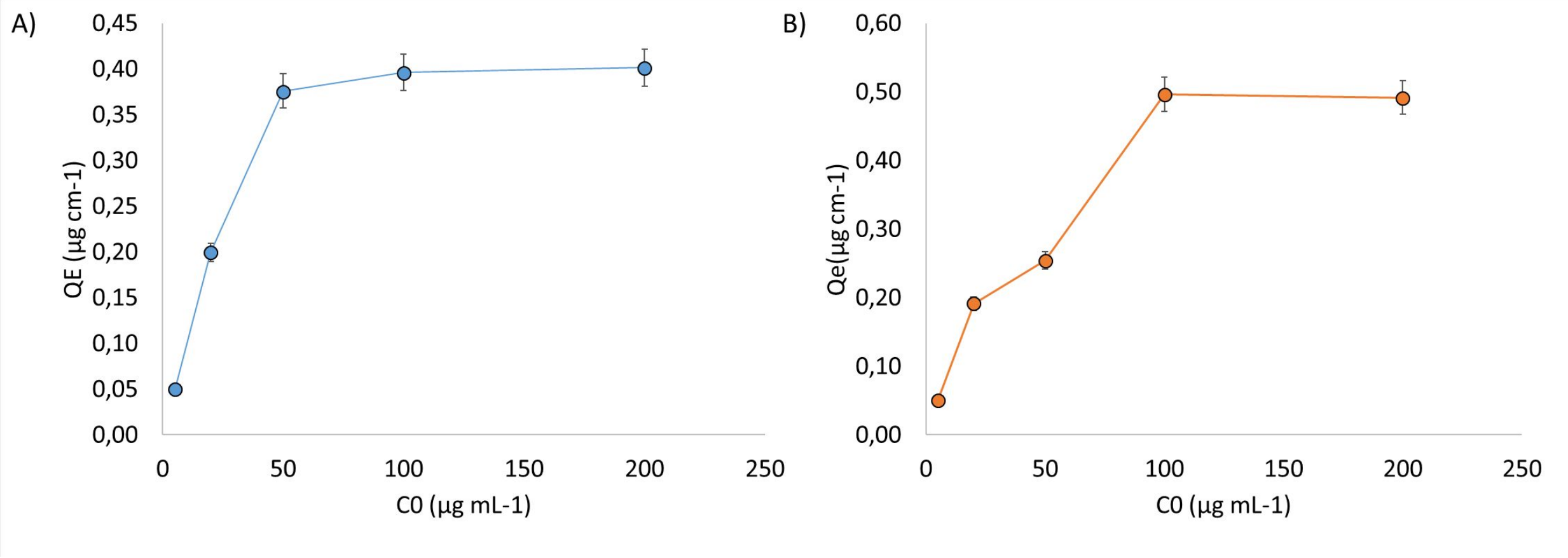

Figure S7: Thermodynamic adsorption of 5-HT (A) and GSH (B) in the TE-PLOT column.

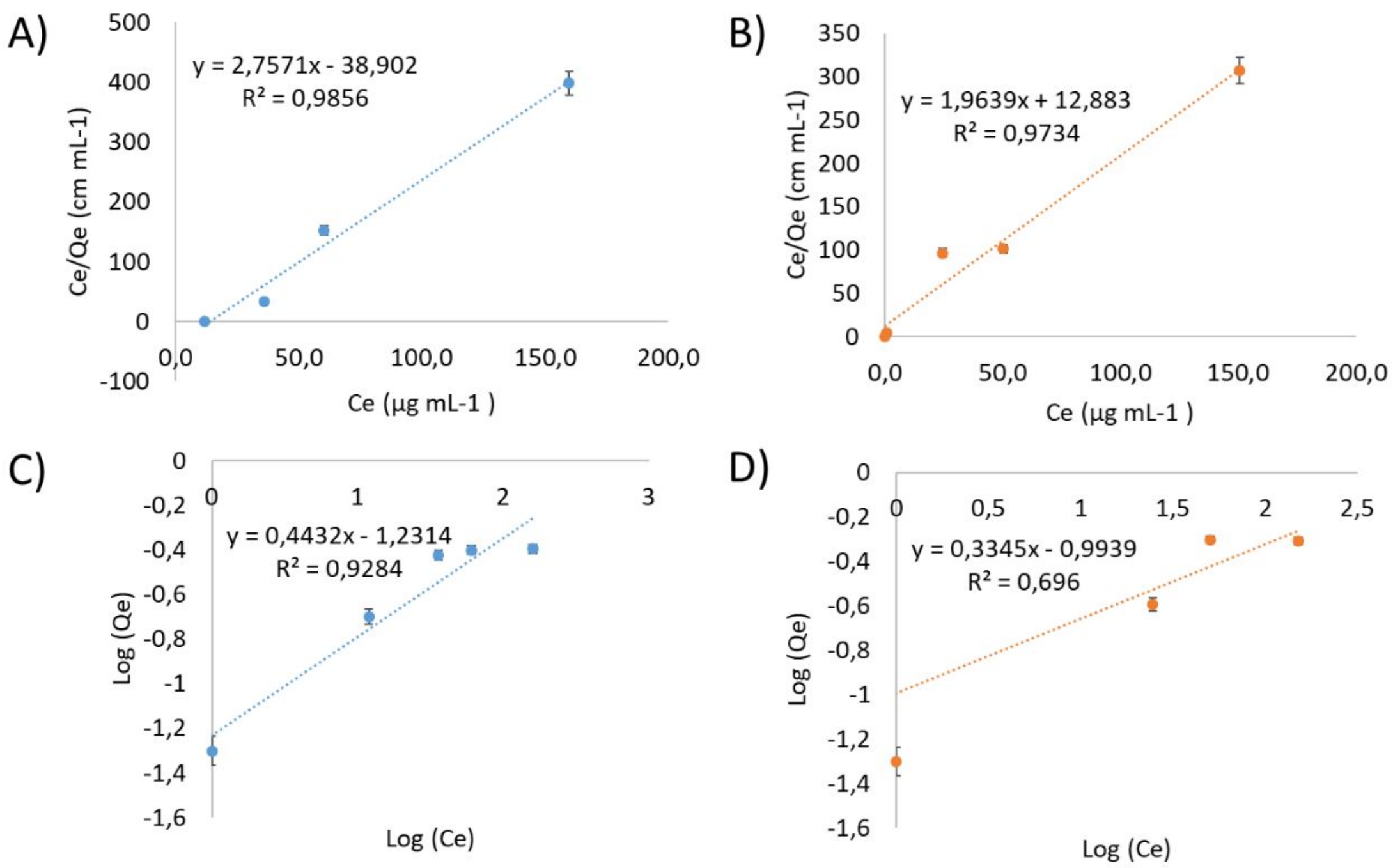

Figure S8: Plots illustrating the application of the Langmuir (A, B) and Freundlich (C, D) models to the experimental rebinding data for 5-HT (A, C) and GSH (B, D). The linear fitting equations and the corresponding coefficients of determination (R^2) are reported at the top of each panel.

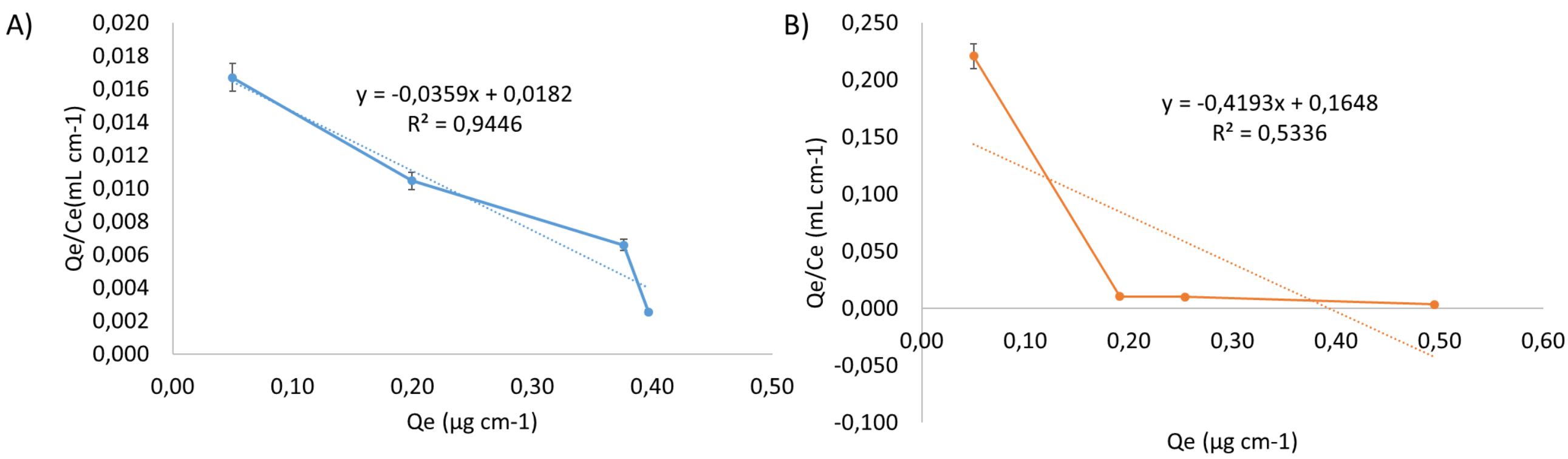

Figure S9: Scatchard fitting of experimental data of the adsorption isotherms, for 5-HT (A), and GSH (B).

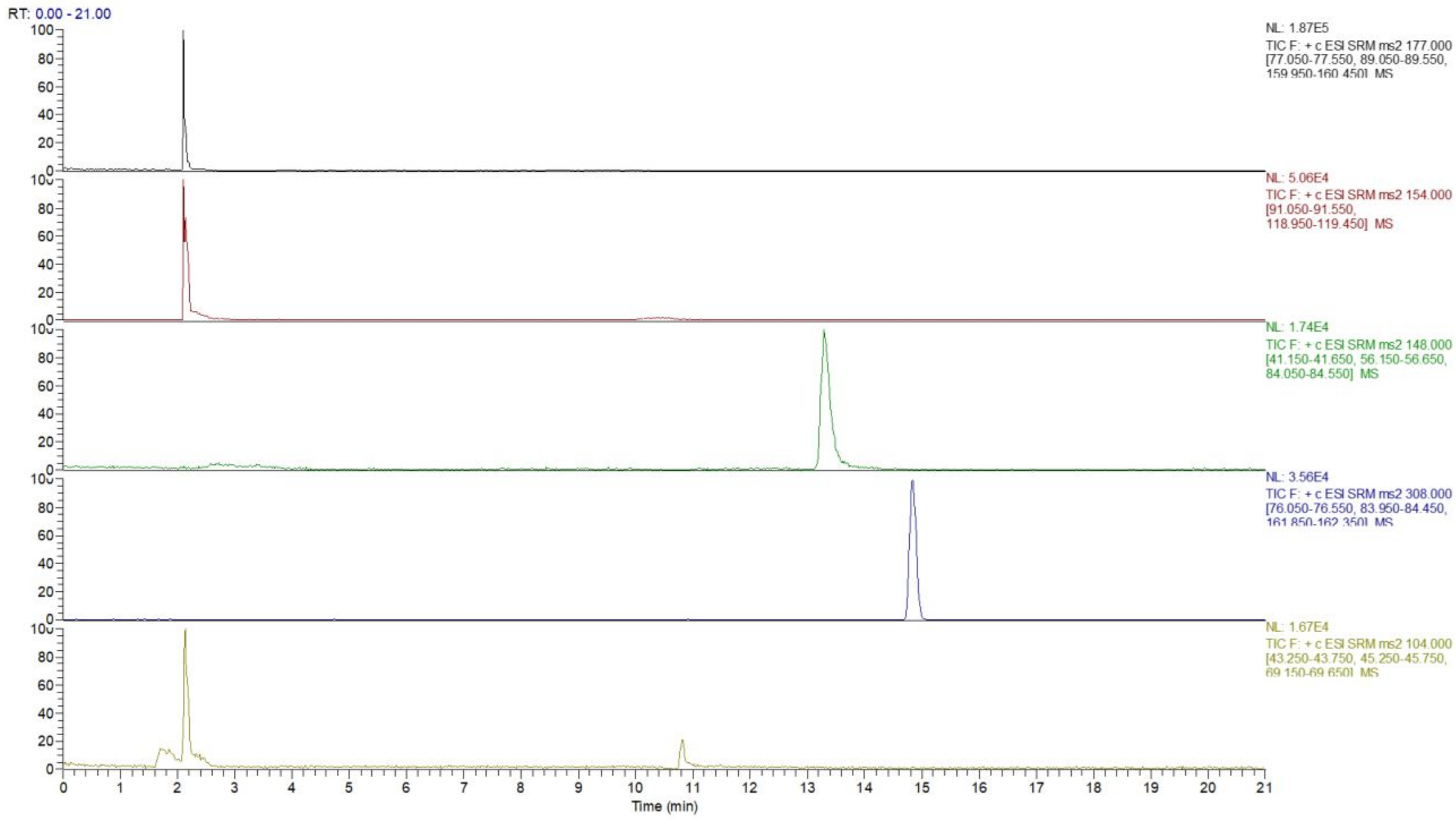

Figura S10: Chromatogram of the plasma sample injected into the in-tube SPME system, showing the endogenous levels of neurotransmitters: from top to bottom, 5-HT (black), DA (red), GLU (green), GSH (blue), and GABA (yellow).

Table S1: Validation results for the plasma matrix at three different concentration levels (C_1: 2.5; C_2: 10; C_3: 60 ng mL⁻¹), linear dynamic range, regression coefficients, LOD, and LOQ.

COMPOUND	RE% (EM%) C_1	RE% (EM%) C_2	RE% (EM%) C_3	R^2	liner range (ng mL^{-1})	LOD (ng mL^{-1})	LOQ (ng mL^{-1})

5-HT	86 (94)	98 (104)	99 (120)	0.97	2.5-100	0.71	2.5
DA	87 (96)	99 (112)	94 (115)	0.98	0.15-100	0.05	0.15
GLU	89 (95)	98 (107)	95 (111)	1.00	1000-10000	0.1	1000
GSH	90 (98)	100 (111)	97 (110)	0.99	2.5-100	0.11	2.5
GABA	99 (102)	100 (97)	99 (98)	0.99	2.5-100	0.14	2.5

Table S2: Validation results for the cell matrix at three different concentration levels (C1: 150; C2: 500; C3: 10000 pmol per 10^6 cells). linear dynamic range. regression coefficients. LOD. and LOQ.

COMPOUND	RE% (EM%) C_1	RE% (EM%) C_2	RE% (EM%) C_3	R^2	liner range (pmol per 10^6 cells)	LOD (pmol per 10^6 cells)	LOQ (pmol per 10^6 cells)
5-HT	95 (108)	97 (110)	99 (112)	1.00	158-10000	20	158
DA	85 (95)	88 (94)	90 (98)	0.99	158-10000	21	158
GLU	102 (105)	105 (108)	108 (108)	0.99	158-10000	5	158
GSH	118 (115)	108 (115)	111 (116)	0.99	158-10000	4	158
GABA	99 (97)	100 (101)	107 (108)	0.99	158-10000	4	158

Table S3: Validation results for the mouse brain matrix at three different concentration levels (C1: 0.03; C2: 0.5; C3: 1.3 μg g⁻¹). linear dynamic range. regression coefficients. LOD. and LOQ.

COMPOUND	RE% (EM%) C_1	RE% (EM%) C_2	RE% (EM%) C_3	R^2	liner range (μg g^{-1})	LOD (μg g^{-1})	LOQ (μg g^{-1})
5-HT	90 (98)	93 (101)	96 (105)	0.99	0.03-1.3	0.01	0.03
DA	89 (97)	89 (96)	89 (95)	0.99	0.03-1.3	0.01	0.03
GLU	105 (101)	108 (104)	105 (106)	1.00	0.03-1.3	0.01	0.03
GSH	108 (112)	104 (110)	115 (112)	1.00	0.03-3.0	0.01	0.03
GABA	96 (99)	99 (101)	101 (105)	1.00	0.01-1.3	0.003	0.01

Table S4: Intraday and interday precision (RSD%) were evaluated by performing recovery experiments (n = 6) on the same day and over six consecutive days at C_2 (10 ng mL⁻¹ for plasma; 500 pmol per 10^6 cells for cells; 0.5 μg g⁻¹ for mouse brain).

	Plasma		Mouse brain		Cells	
COMPOUND	Intra-day (RSD %)	Inter-day (RSD %)	Intra-day (RSD %)	Inter-day (RSD %)	Intra-day (RSD %)	Inter-day (RSD %)
5-HT	5	4	6	4	6	5
DA	7	5	6	4	7	5
GLU	5	4	7	5	6	4

GSH	6	4	7	5	6	3
GABA	5	4	6	5	7	5

Table S5: Recovery values and matrix effect for different biological matrices compared with and without clean-up. Spiking level for plasma: 2.5 ng mL⁻¹; for cells: 150 pmol per 10^6 cells; for mouse brain: 0.5 µg g⁻¹.

	Plasma				Mouse brain				Cells			
	no-clean up		in-tube SPME		no-clean up		in-tube SPME		no-clean up		in-tube SPME	
	RE%	ME%	RE%	ME%	RE%	ME%	RE%	ME%	RE%	ME%	RE%	ME%
5-HT	n.d	75±3	86±5	94±8	90±8	130±6	90±6	98±5	95±8	130±10	95±6	108±3
DA	n.d	70±5	87±6	96±5	89±9	140±7	89±6	97±2	85±7	120±9	85±6	95±2
GLU	65±6	150±4	89±5	95±5	104±7	150±8	105±8	101±2	100±8	150±11	102±7	105±3
GSH	70±6	140±8	90±6	98±5	108±10	140±11	108±8	112±3	110±8	120±15	118±7	115±3
GABA	64±6	145±4	99±5	102±6	96±10	150±15	96±6	99 ± 2	98±8	120±13	99±7	97 ± 2

Table S6: Diagnostic MS/MS fragmentation data for the tentatively identified metabolites in positive mode.

Name	Formula	Calc. Molecular Weight	Exp. Molecular Weight	Calc. m/z	Exp. m/z	Δmass (ppm)	RT [min]	Main product ions
Testosterone sulfate	C19H28O5S	368.165747	368.16597	367.157922	367.158145	-0.61	11.345	367.1583, 96.9598, 299.1500
Xylenesulfonate	C8H10O3S	186.035067	186.0351	185.027242	185.027275	-0.18	11.973	185.0276, 170.0043, 121.0658, 79.9568
PC 16:0_20:4	C44H80NO8P	827.567637	827.5686	826.559812	826.560775	-1.17	12.063	766.5395, 303.2332, 255.2331, 224.0694, 168.0431, 152.9958, 78.9586
PC 18:0_18:2	C44H84NO8P	831.598937	831.5996	830.591112	830.591775	-0.80	12.122	770.5730, 508.3407, 279.2330, 283.2643, 224.0694, 168.0431, 152.9958, 78.9586
PC 16:0_18:2	C42H80NO8P	803.567637	803.56843	802.559812	802.560605	-0.99	12.145	742.5396, 279.2330, 255.2330, 168.0430, 78.9585
PC 16:0_18:1	C42H82NO8P	805.583287	805.58375	804.575462	804.575925	-0.58	12.154	744.5545, 281.2486, 255.2330, 224.0694, 168.0431, 152.9958, 78.9586
Arginine	C6H14N4O2	174.111676	174.11172	173.103851	173.103895	-0.25	12.347	173.1047, 131.0825, 129.1033, 116.0717
Histidine	C6H9N3O2	155.069477	155.06956	154.061652	154.061735	-0.54	12.41	154.0622, 137.0356, 110.0724, 93.0455
Tryptophan	C11H12N2O2	204.089878	204.09005	203.082053	203.082225	-0.85	12.448	203.0826, 159.0926, 142.0661, 116.0505, 74.0242
LPE 20:4	C25H44NO7P	501.285542	501.28611	500.277717	500.278285	-1.14	12.468	500.2784, 303.2331, 196.0380, 140.0118, 78.9586
LPE 18:2	C30H39NO4	477.287909	477.28572	476.280084	476.277895	4.60	12.53	476.2782, 279.2330, 196.0380, 140.0118, 78.9586
LPE 16:0	C20H43N3O6S	453.287259	453.28599	452.279434	452.278165	2.81	12.564	452.2783, 255.2330, 196.0382, 140.0119, 78.9586
Phenylalanine	C9H11NO2	165.078979	165.07911	164.071154	164.071285	-0.80	12.569	164.0716, 147.0451, 72.0085
SM 34:1	C39H79N2O6P	748.573056	748.5738	747.565231	747.565975	-1.00	12.663	687.5441, 168.0430, 78.9585
Uric Acid	C5H4N4O3	168.028341	168.02832	167.020516	167.020495	0.13	12.726	167.0210, 124.0151, 96.0201, 69.0089
Valine	C5H11NO2	117.078979	117.07902	116.071154	116.071195	-0.35	12.769	116.0716, 88.0116

Tyrosine	C9H11NO3	181.073894	181.07394	180.066069	180.066115	-0.26	12.792	180.0666, 163.0401, 119.0502, 93.0343, 72.0086
LPC 18:0	C26H54NO7P	509.348142	509.34845	508.340317	508.340625	-0.61	12.949	508.3406, 283.2644, 224.0694, 168.0431, 152.9958, 78.9586
LPC 20:4	C28H50NO7P	589.337972	589.33853	588.330147	588.330705	-0.95	12.987	528.3095, 303.2331, 224.0694, 168.0431, 152.9958, 78.9586
LPC 18:1	C26H52NO7P	507.332492	507.333	506.324667	506.325175	-1.00	13.016	506.3250, 281.2487, 224.0694, 168.0430, 152.9959, 78.9586
LPC 18:2	C26H50NO7P	505.316842	505.31707	504.309017	504.309245	-0.45	13.08	504.3097, 279.2330, 224.0694, 168.0649, 152.9960, 78.9585
LPC 16:0	C24H50NO7P	481.316842	481.31719	480.309017	480.309365	-0.72	13.114	480.3097, 283.2642, 255.2329, 224.0695, 78.9585
Taurine	C2H7NO3S	125.014666	125.01465	124.006841	124.006825	0.13	13.224	124.0073, 79.9569
Sucrose	C12H22O11	342.116215	342.11653	341.10839	341.108705	-0.92	13.303	341.1089, 179.0560, 101.0242, 89.0240, 59.0132, 71.0133
Threonine	C4H9NO3	119.058244	119.05831	118.050419	118.050485	-0.56	13.531	118.0509, 74.0242, 72.0085
Serine	C3H7NO3	105.042594	105.04251	104.034769	104.034685	0.81	13.669	104.0351, 74.0242, 72.0085
Glutamine	C5H10N2O3	146.069143	146.06913	145.061318	145.061305	0.09	13.808	145.0618, 127.0612, 109.0407, 84.0451, 74.0242, 58.0292
Threonic Acid	C4H8O5	136.037175	136.03726	135.02935	135.029435	-0.63	13.828	135.0299, 117.0193, 89.0240, 75.0082, 59.0132
Phenylacetylglutamine	C13H16N2O4	264.111008	264.11122	263.103183	263.103395	-0.81	13.892	263.1035, 145.618, 127.0512, 109.0407, 84.0451
Ornithine	C5H12N2O2	132.089878	132.08986	131.082053	131.082035	0.14	13.91	131.0825, 114.0196, 113.0357, 70.0293
Citrulline	C6H13N3O3	175.095692	175.09562	174.087867	174.087795	0.41	13.914	174.0884, 131.0825, 87.0083, 114.0197
Glycerophosphoethanolamine	C5H14NO6P	215.055877	215.05611	214.048052	214.048285	-1.09	15.137	241.0487, 140.0118, 78.9586
Cystine	C6H12N2O4S2	240.023852	240.02405	239.016027	239.016225	-0.83	15.736	239.0164, 151.9845, 120.0124, 74.0065, 86.0244
Glycerophosphocholine	C7H18NO6P	243.087177	243.08746	242.079352	242.079635	-1.17	15.85	242.0801, 168.0430, 78.9586
gamma-Aminobutyric acid	C4H9NO2	103.063329	103.06324	102.055504	102.055415	0.87	15.948	102.0559. 84.0450
Glutamic acid	C5H9NO4	147.053159	147.0532	146.045334	146.045375	-0.28	15.976	146.0459, 128.0353, 102.0559, 85.0291

Table S7: Diagnostic MS/MS fragmentation data for the tentatively identified metabolites in negative mode.

Name	Formula	Calc. Molecular Weight	Exp. Molecular Weight	Calc. m/z	Exp. m/z	Δmass (ppm)	RT [min]	Main product ions
Testosterone sulfate	C19H28O5S	368.165747	368.16597	367.157922	367.158145	-0.61	11.345	367.1583, 96.9598, 299.1500
Xylenesulfonate	C8H10O3S	186.035067	186.0351	185.027242	185.027275	-0.18	11.973	185.0276, 170.0043, 121.0658, 79.9568
PC 16:0_20:4	C44H80NO8P	827.567637	827.5686	826.559812	826.560775	-1.17	12.063	766.5395, 303.2332, 255.2331, 224.0694, 168.0431, 152.9958, 78.9586
PC 18:0_18:2	C44H84NO8P	831.598937	831.5996	830.591112	830.591775	-0.80	12.122	770.5730, 508.3407, 279.2330, 283.2643, 224.0694, 168.0431, 152.9958, 78.9586
PC 16:0_18:2	C42H80NO8P	803.567637	803.56843	802.559812	802.560605	-0.99	12.145	742.5396, 279.2330, 255.2330, 168.0430, 78.9585
PC 16:0_18:1	C42H82NO8P	805.583287	805.58375	804.575462	804.575925	-0.58	12.154	744.5545, 281.2486, 255.2330, 224.0694, 168.0431, 152.9958, 78.9586
Arginine	C6H14N4O2	174.111676	174.11172	173.103851	173.103895	-0.25	12.347	173.1047, 131.0825, 129.1033, 116.0717
Histidine	C6H9N3O2	155.069477	155.06956	154.061652	154.061735	-0.54	12.41	154.0622, 137.0356, 110.0724, 93.0455
Tryptophan	C11H12N2O2	204.089878	204.09005	203.082053	203.082225	-0.85	12.448	203.0826, 159.0926, 142.0661, 116.0505, 74.0242
LPE 20:4	C25H44NO7P	501.285542	501.28611	500.277717	500.278285	-1.14	12.468	500.2784, 303.2331, 196.0380, 140.0118, 78.9586
LPE 18:2	C30H39NO4	477.287909	477.28572	476.280084	476.277895	4.60	12.53	476.2782, 279.2330, 196.0380, 140.0118, 78.9586
LPE 16:0	C20H43N3O6S	453.287259	453.28599	452.279434	452.278165	2.81	12.564	452.2783, 255.2330, 196.0382, 140.0119, 78.9586
Phenylalanine	C9H11NO2	165.078979	165.07911	164.071154	164.071285	-0.80	12.569	164.0716, 147.0451, 72.0085
SM 34:1	C39H79N2O6P	748.573056	748.5738	747.565231	747.565975	-1.00	12.663	687.5441, 168.0430, 78.9585
Uric Acid	C5H4N4O3	168.028341	168.02832	167.020516	167.020495	0.13	12.726	167.0210, 124.0151, 96.0201, 69.0089
Valine	C5H11NO2	117.078979	117.07902	116.071154	116.071195	-0.35	12.769	116.0716, 88.0116
Tyrosine	C9H11NO3	181.073894	181.07394	180.066069	180.066115	-0.26	12.792	180.0666, 163.0401, 119.0502, 93.0343, 72.0086
LPC 18:0	C26H54NO7P	509.348142	509.34845	508.340317	508.340625	-0.61	12.949	508.3406, 283.2644, 224.0694, 168.0431, 152.9958, 78.9586
LPC 20:4	C28H50NO7P	589.337972	589.33853	588.330147	588.330705	-0.95	12.987	528.3095, 303.2331, 224.0694, 168.0431, 152.9958, 78.9586
LPC 18:1	C26H52NO7P	507.332492	507.333	506.324667	506.325175	-1.00	13.016	506.3250, 281.2487, 224.0694, 168.0430, 152.9959, 78.9586

LPC 18:2	C26H50NO7P	505.316842	505.31707	504.309017	504.309245	-0.45	13.08	504.3097, 279.2330, 224.0694, 168.0649, 152.9960, 78.9585
LPC 16:0	C24H50NO7P	481.316842	481.31719	480.309017	480.309365	-0.72	13.114	480.3097, 283.2642, 255.2329, 224.0695, 78.9585
Taurine	C2H7NO3S	125.014666	125.01465	124.006841	124.006825	0.13	13.224	124.0073, 79.9569
Sucrose	C12H22O11	342.116215	342.11653	341.10839	341.108705	-0.92	13.303	341.1089, 179.0560, 101.0242, 89.0240, 59.0132, 71.0133
Threonine	C4H9NO3	119.058244	119.05831	118.050419	118.050485	-0.56	13.531	118.0509, 74.0242, 72.0085
Serine	C3H7NO3	105.042594	105.04251	104.034769	104.034685	0.81	13.669	104.0351, 74.0242, 72.0085
Glutamine	C5H10N2O3	146.069143	146.06913	145.061318	145.061305	0.09	13.808	145.0618, 127.0612, 109.0407, 84.0451, 74.0242, 58.0292
Threonic Acid	C4H8O5	136.037175	136.03726	135.02935	135.029435	-0.63	13.828	135.0299, 117.0193, 89.0240, 75.0082, 59.0132
Phenylacetylglutamine	C13H16N2O4	264.111008	264.11122	263.103183	263.103395	-0.81	13.892	263.1035, 145.618, 127.0512, 109.0407, 84.0451
Ornithine	C5H12N2O2	132.089878	132.08986	131.082053	131.082035	0.14	13.91	131.0825, 114.0196, 113.0357, 70.0293
Citrulline	C6H13N3O3	175.095692	175.09562	174.087867	174.087795	0.41	13.914	174.0884, 131.0825, 87.0083, 114.0197
Glycerophosphoethanolamine	C5H14NO6P	215.055877	215.05611	214.048052	214.048285	-1.09	15.137	241.0487, 140.0118, 78.9586
Cystine	C6H12N2O4S2	240.023852	240.02405	239.016027	239.016225	-0.83	15.736	239.0164, 151.9845, 120.0124, 74.0065, 86.0244
Glycerophosphocholine	C7H18NO6P	243.087177	243.08746	242.079352	242.079635	-1.17	15.85	242.0801, 168.0430, 78.9586
gamma-Aminobutyric acid	C4H9NO2	103.063329	103.06324	102.055504	102.055415	0.87	15.948	102.0559. 84.0450
Glutamic acid	C5H9NO4	147.053159	147.0532	146.045334	146.045375	-0.28	15.976	146.0459, 128.0353, 102.0559, 85.0291
